# Supplementary material for: Structural bioinformatics studies of serotonin, dopamine and norepinephrine transporters and their AlphaFold2 predicted water-soluble QTY variants and uncovering the natural mutations of L->Q, I->T, F->Y and Q->L, T->I and Y->F
Source: PLoS One. 2024 Mar 22;19(3):e0300340. doi: 10.1371/journal.pone.0300340 (PMC10959339; doi:10.1371/journal.pone.0300340)
Supplement: S1 File — (DOCX) [file pone.0300340.s001.docx]

**Supplementary Information**

**Structural bioinformatics studies of serotonin, dopamine and norepinephrine transporters and their AlphaFold2 predicted water-soluble QTY variants and uncovering the natural mutations of L->Q, I->T, F->Y and Q->L, T->I and Y->F**

Taner Karagöl^1,¶^, Alper Karagöl^1,¶^, Shuguang Zhang^2,*^

^1^Istanbul University Istanbul Medical Faculty, Istanbul, Turkey

^2^Laboratory of Molecular Architecture, Media Lab, Massachusetts Institute of Technology, 77 Massachusetts Avenue, Cambridge, MA, 02139, USA

^¶^These authors contribute equally.

*To whom the correspondence should be addressed.

Email:

Taner Karagöl, [taner.karagol@gmail.com](mailto:taner.karagol@gmail.com) ORCID: [0009-0005-1011-7661](https://orcid.org/0009-0005-1011-7661)

Alper Karagöl, [alper.karagol@gmail.com](mailto:alper.karagol@gmail.com) ORCID: [0009-0001-7864-0732](https://orcid.org/0009-0001-7864-0732)

Shuguang Zhang, [Shuguang@MIT.EDU](mailto:Shuguang@MIT.EDU) ORCID: [0000-0002-3856-3752](https://orcid.org/0000-0002-3856-3752)

**Supplementary Figures**

**Figure S1**


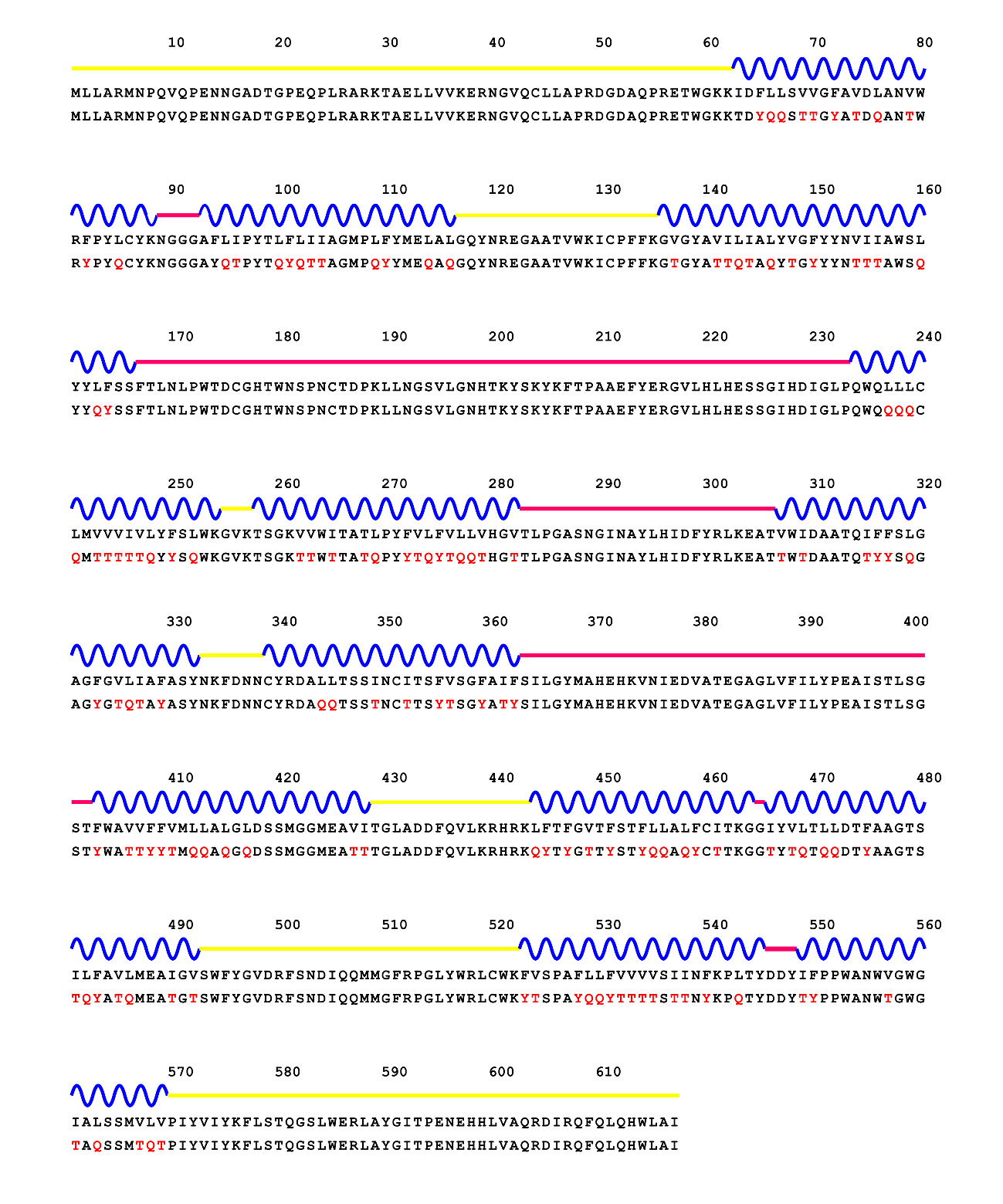


**a**, NET


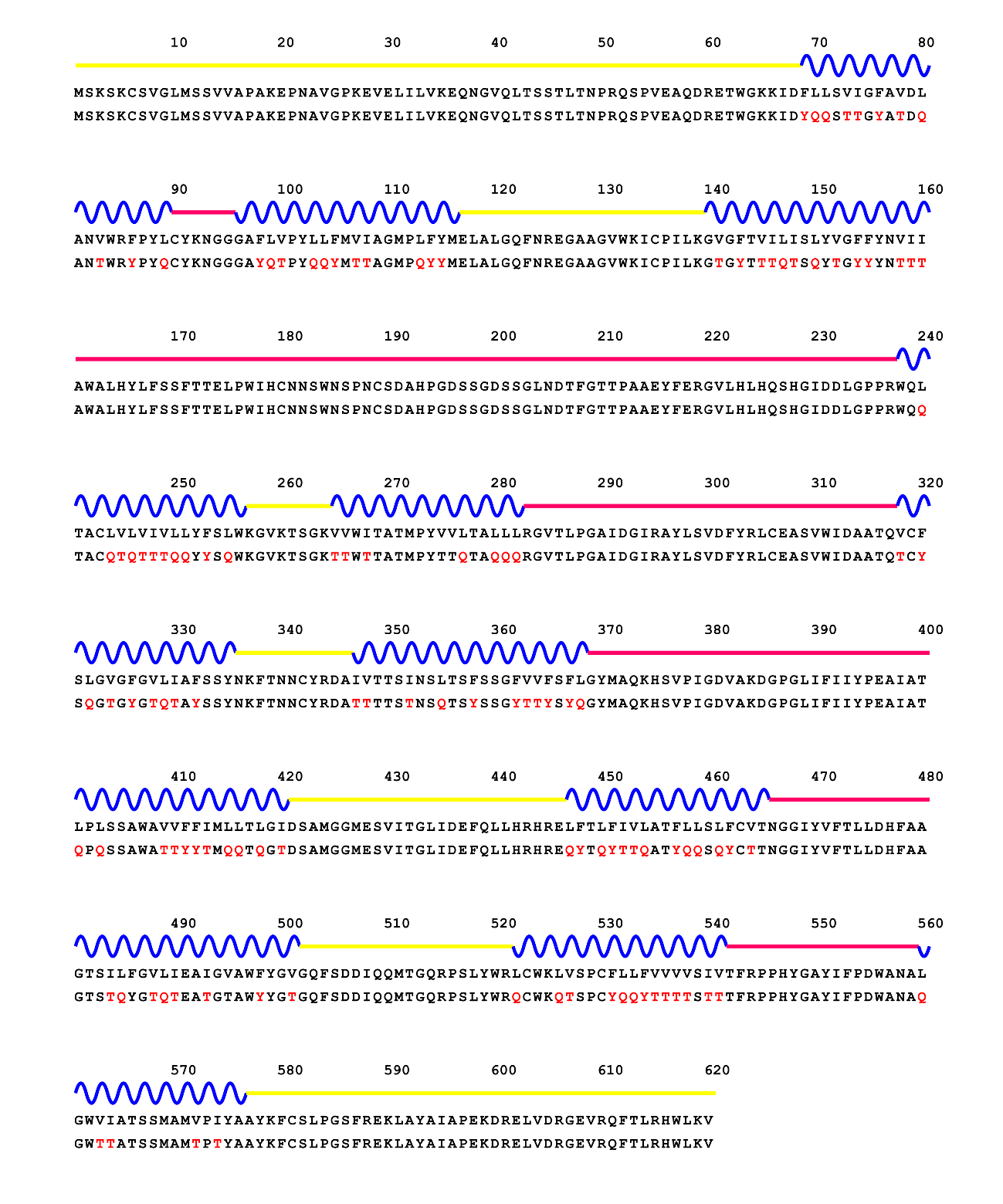


**b**, DAT


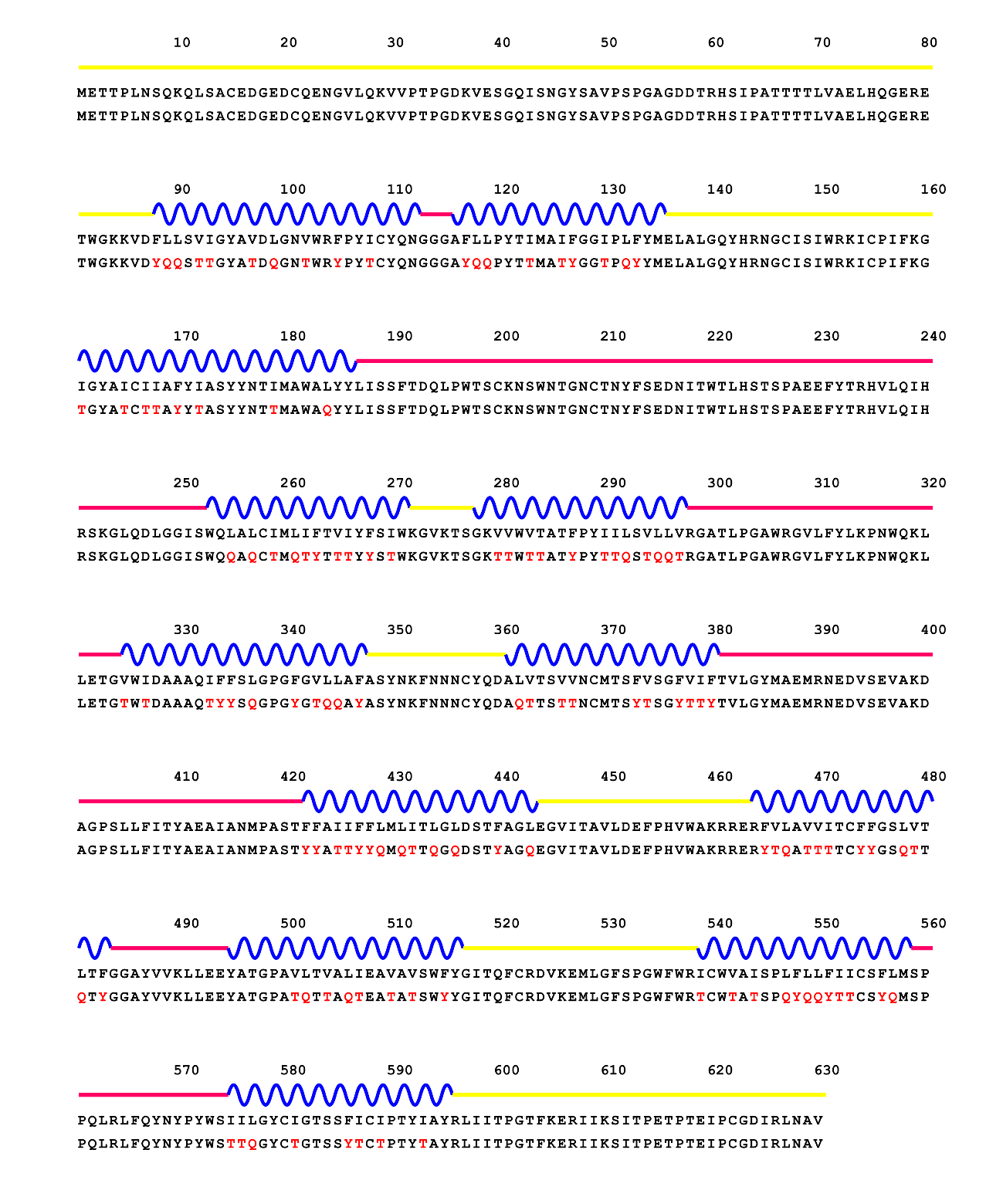


**c**, SERT


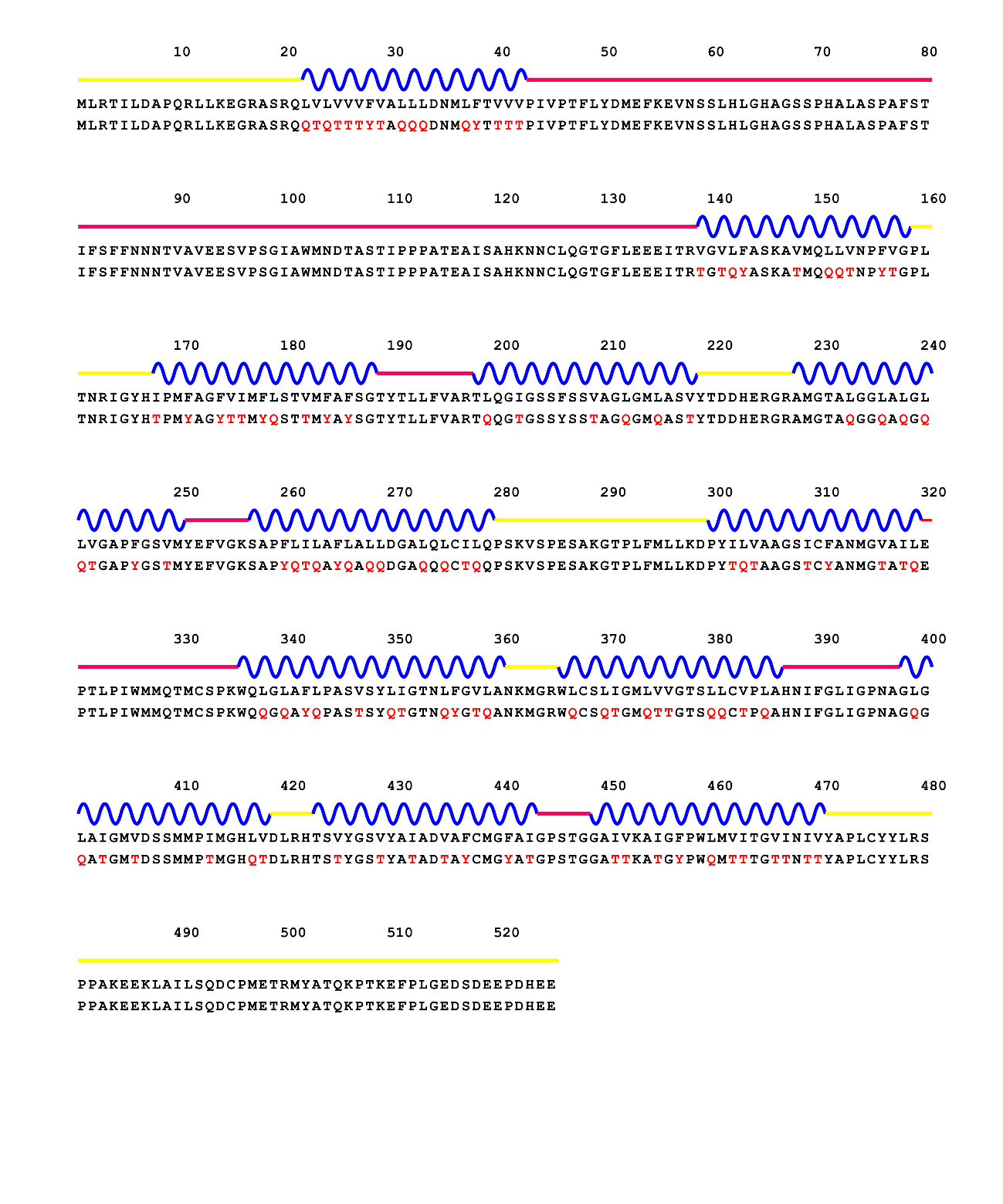


**d**, VMAT1


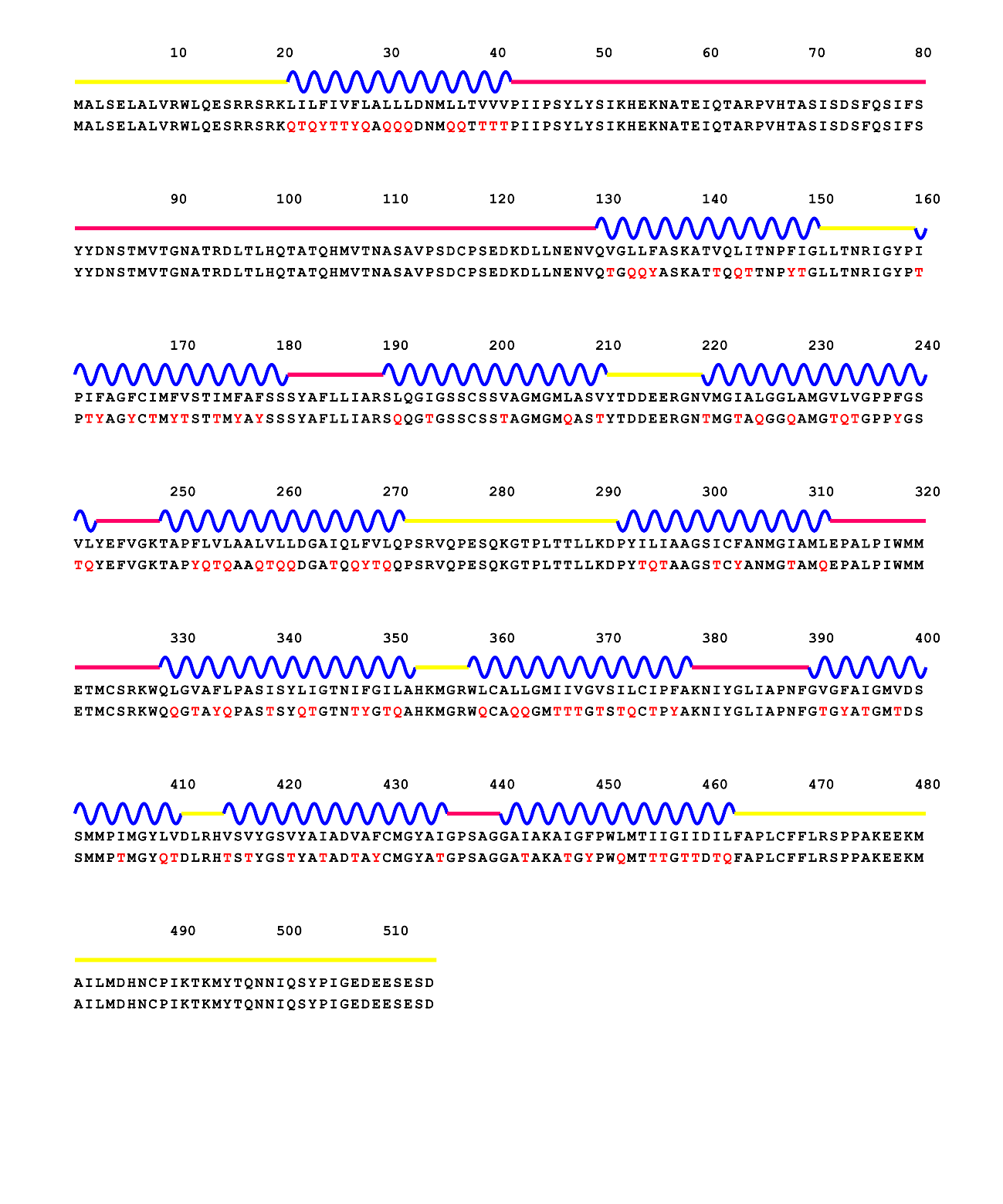


**e**, VMAT2


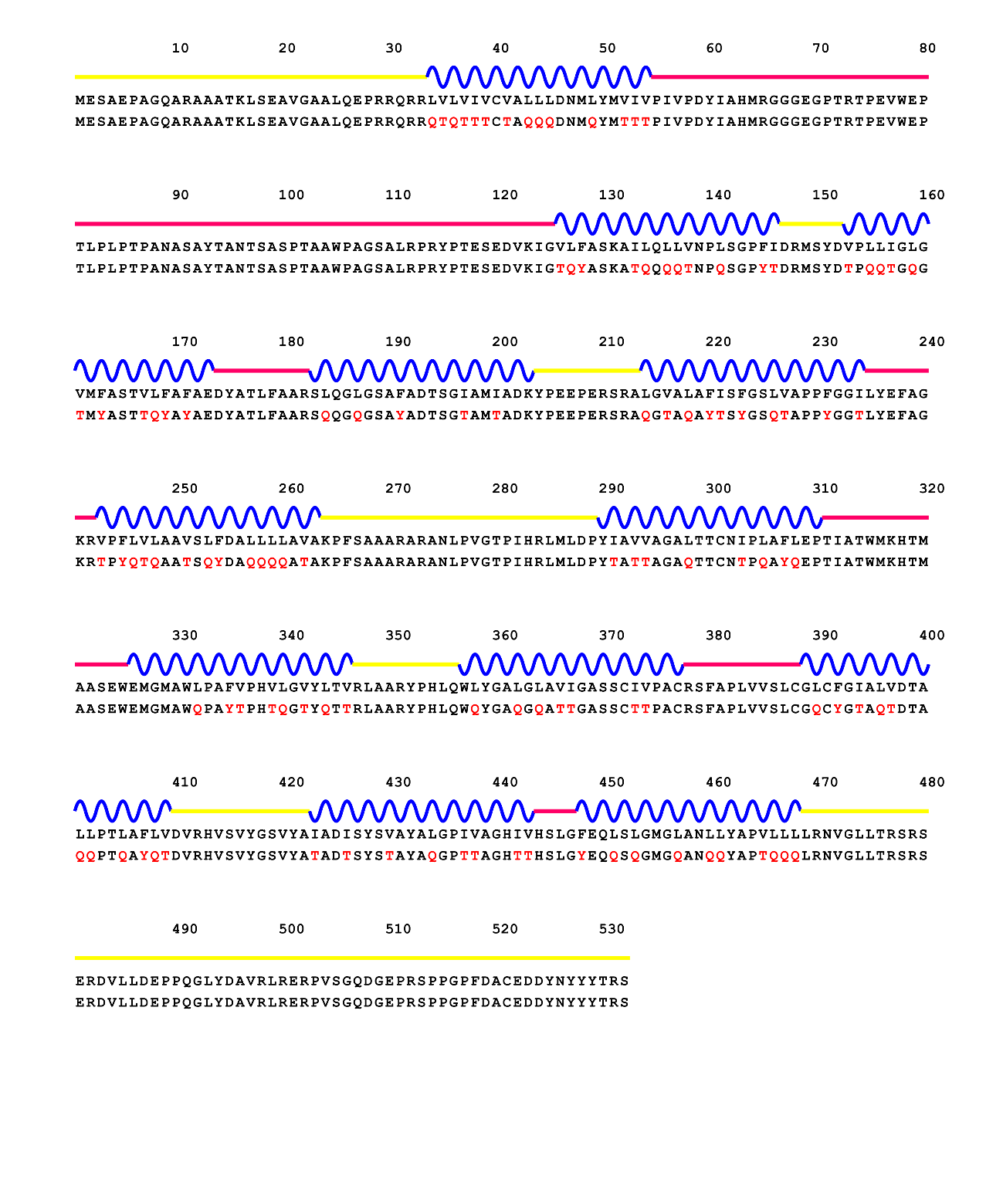


**f**, VAChT


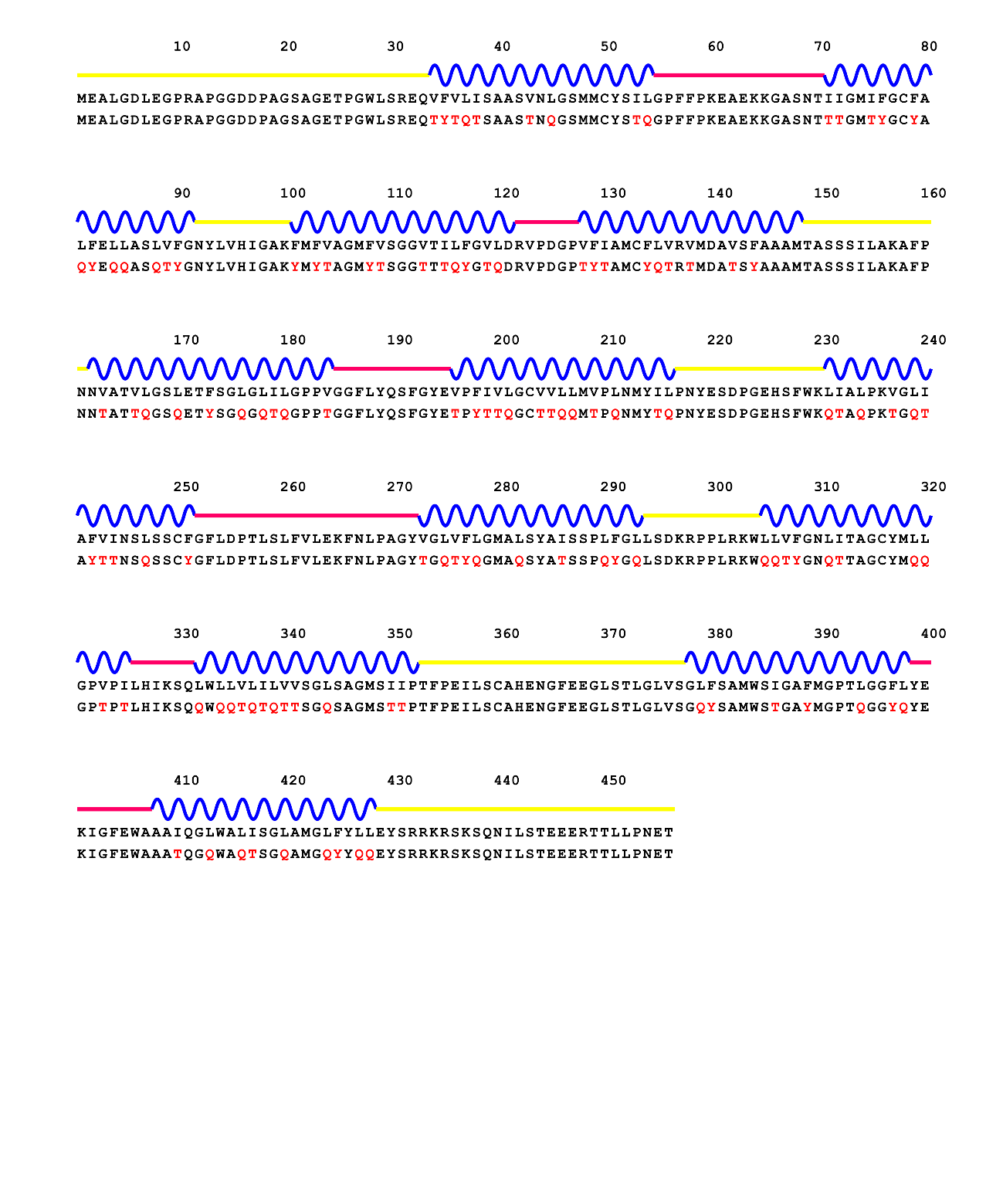


**g**, VPAT

**Figure S1. Enlarged panel a-g of Figure 1.** The protein sequences of each monoamine transporter are now clearly visible. The QTY variant sequences are below the native protein sequences. The QTY amino acid substitution changes are colored in red. Other colour code: Yellow line-intracellular, Blue wave-transmembrane helices, Pinkish line-extracellular, Green line-peripheral domains and hairpin loops.


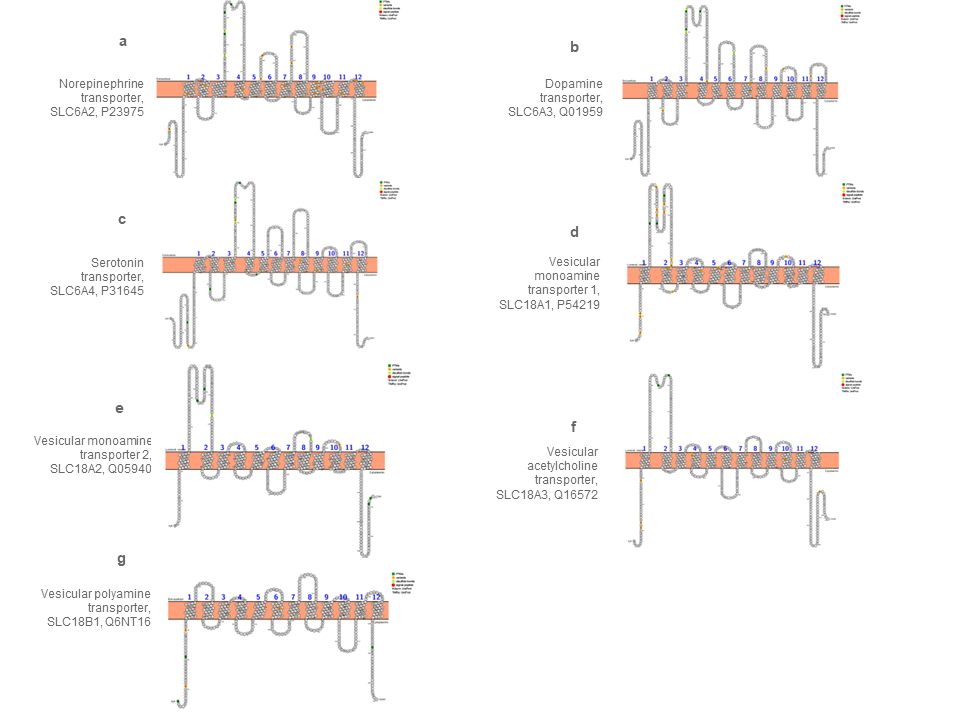


**Figure S2. Membrane topology models of seven transporters.** Prediction was performed with the Protter web client, based on the sequence analysis of the corresponding transporter using the UniProt database. Each transporter has a 12-transmembrane (TM) architecture.

**
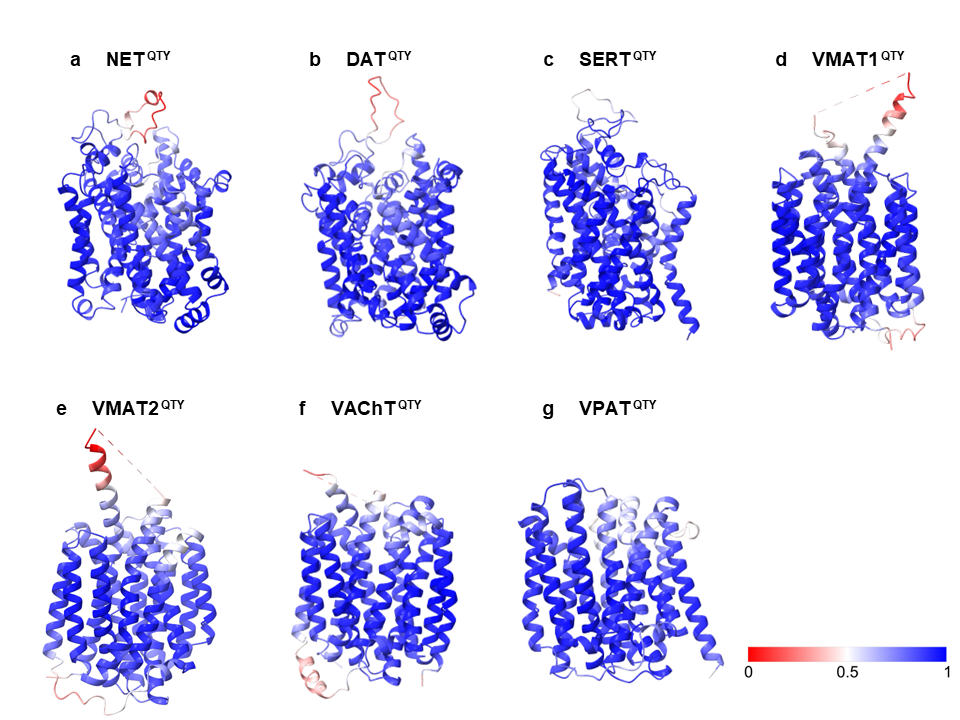
**

**Figure S3. Prediction confidence of AlphaFold2 models of the QTY variants.** The structures are colored according to predicted Local Distance Difference Test (pLDDT) scores for assessing local structure confidence, with the scale shown at the bottom right.

**
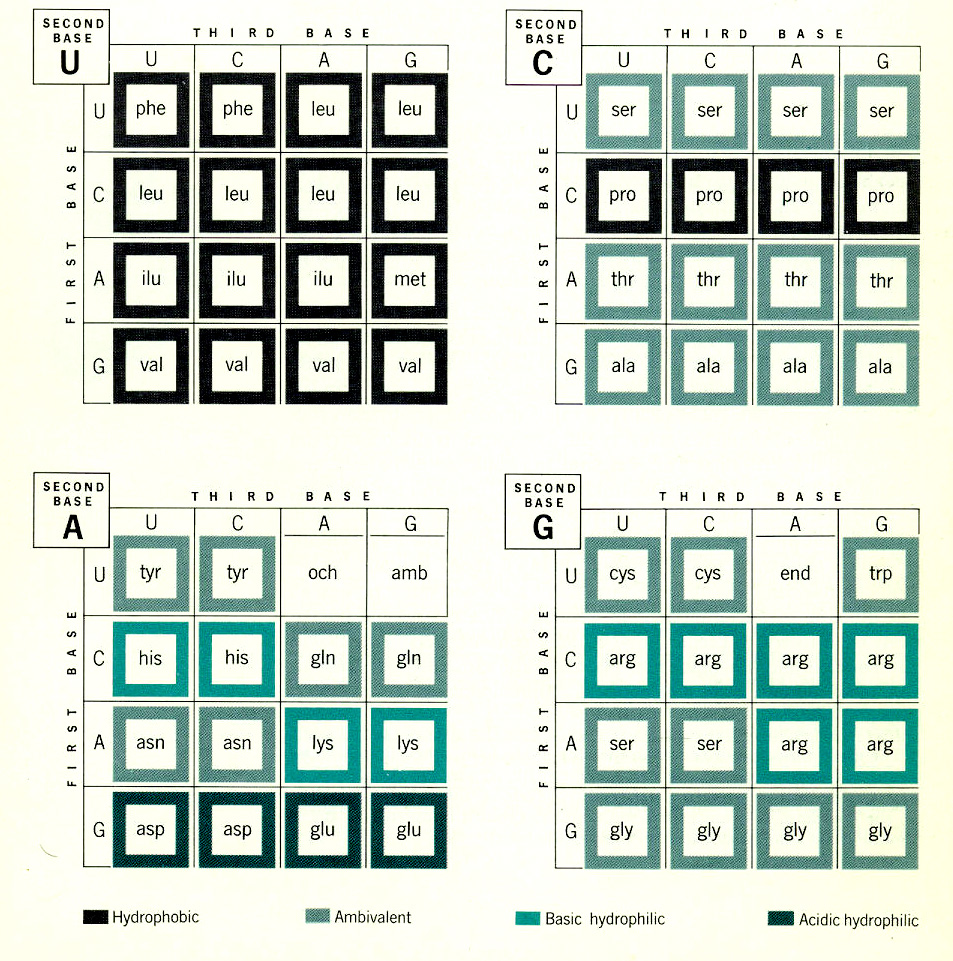
**

**Figure S4. The second position importance of Genetic code.** The Genetic code’s second position determines the chemical nature of amino acids. Their second position is emphasized. For example, ***i***) amino acids with U at the second position are hydrophobic (Phe, Leu, Ile, Val and Met); ***ii***) amino acids with C at the second position are less hydrophobic (Pro and Ala), or with a hydroxyl OH group (Ser and Thr); ***iii***) amino acids with A at the second position are hydrophilic and water soluble (Asp, Glu, Asn, Glu, Lys, His and Tyr), and 2 stop codons Ochre (UAA) and Amber (UAG); ***iv***) amino acids (Arg and Ser) with G at the second position are water soluble, Cys is partially water-soluble and Gly is achiral and has a H as the side chain. The stop codon is UGA. In general, pyrimidine U and C at the second position confer hydrophobicity; in contrast, purine A and G at the second position confer hydrophilicity. (This Figure S4 is adopted from the book of Proteins by Richard Dickerson, 1968, Harper Collins).


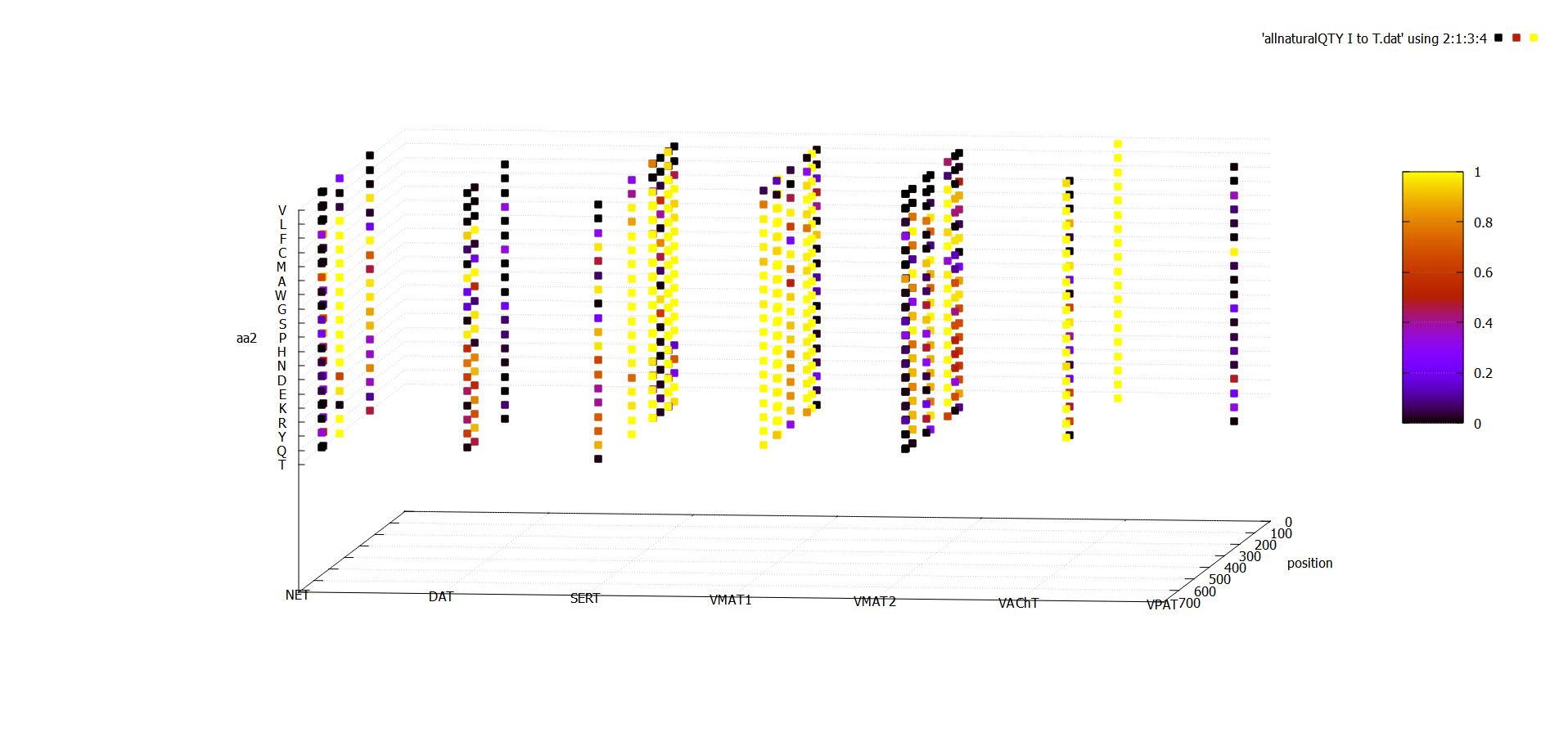


**Figure S5. Predicted effects of variations at the residue where natural I to T mutations observed.** The names of the transporters are listed on the x-axis. The z-axis indicates the position of the substitution within the native protein sequence, while the color scale represent the PolyPhen-2 predicted effect of the substitution, ranging from benign (0.0) to damaging (1.0). The y-axis indicates the second amino acid that replaced the wild-type (I) amino acid residue.


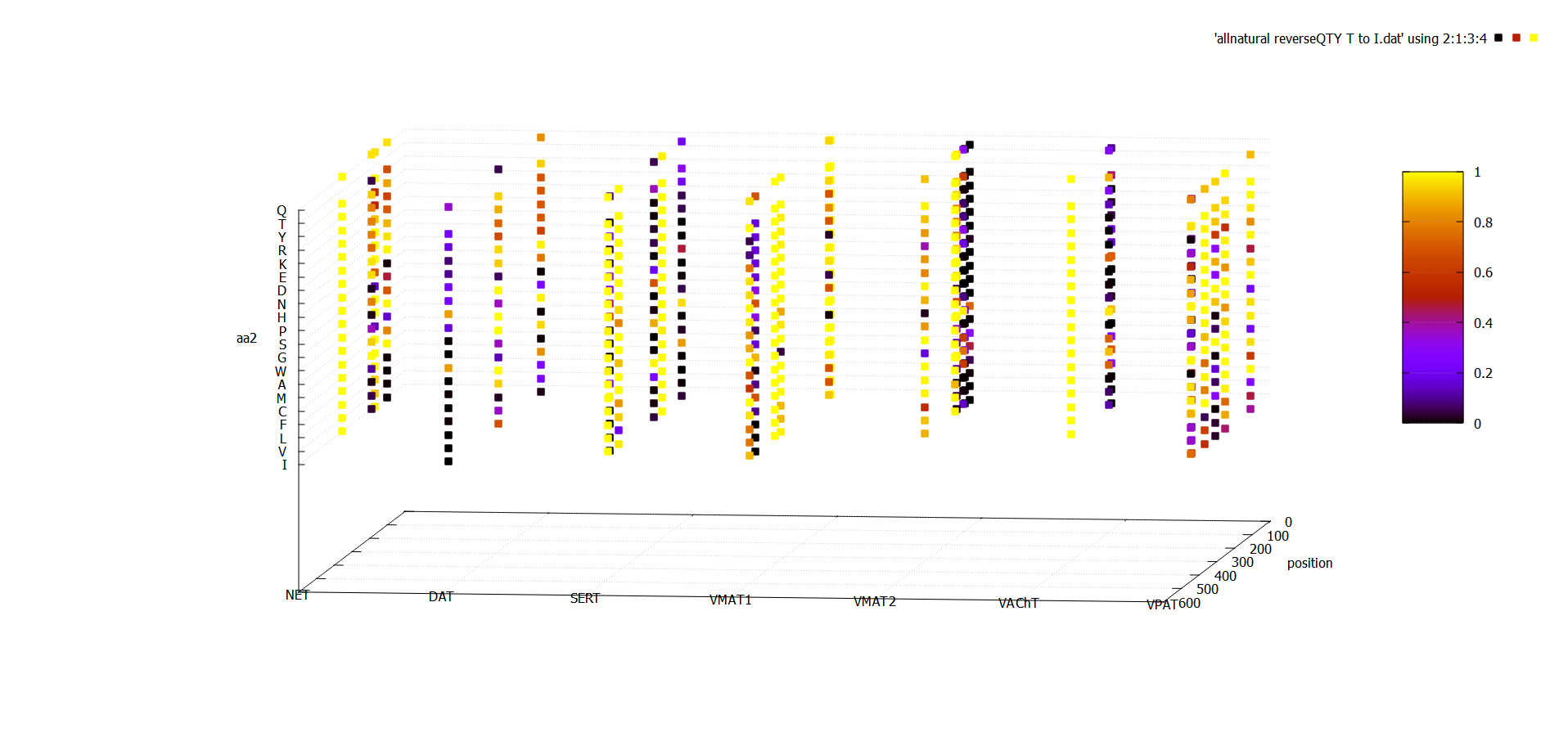


**Figure S6. Predicted effects of variations at the residue where natural T to I mutations observed.** The names of the transporters are listed on the x-axis. The z-axis indicates the position of the substitution within the native protein sequence, while the color scale represent the PolyPhen-2 predicted effect of the substitution, ranging from benign (0.0) to damaging (1.0). The y-axis indicates the second amino acid that replaced the wild-type (T) amino acid residue.


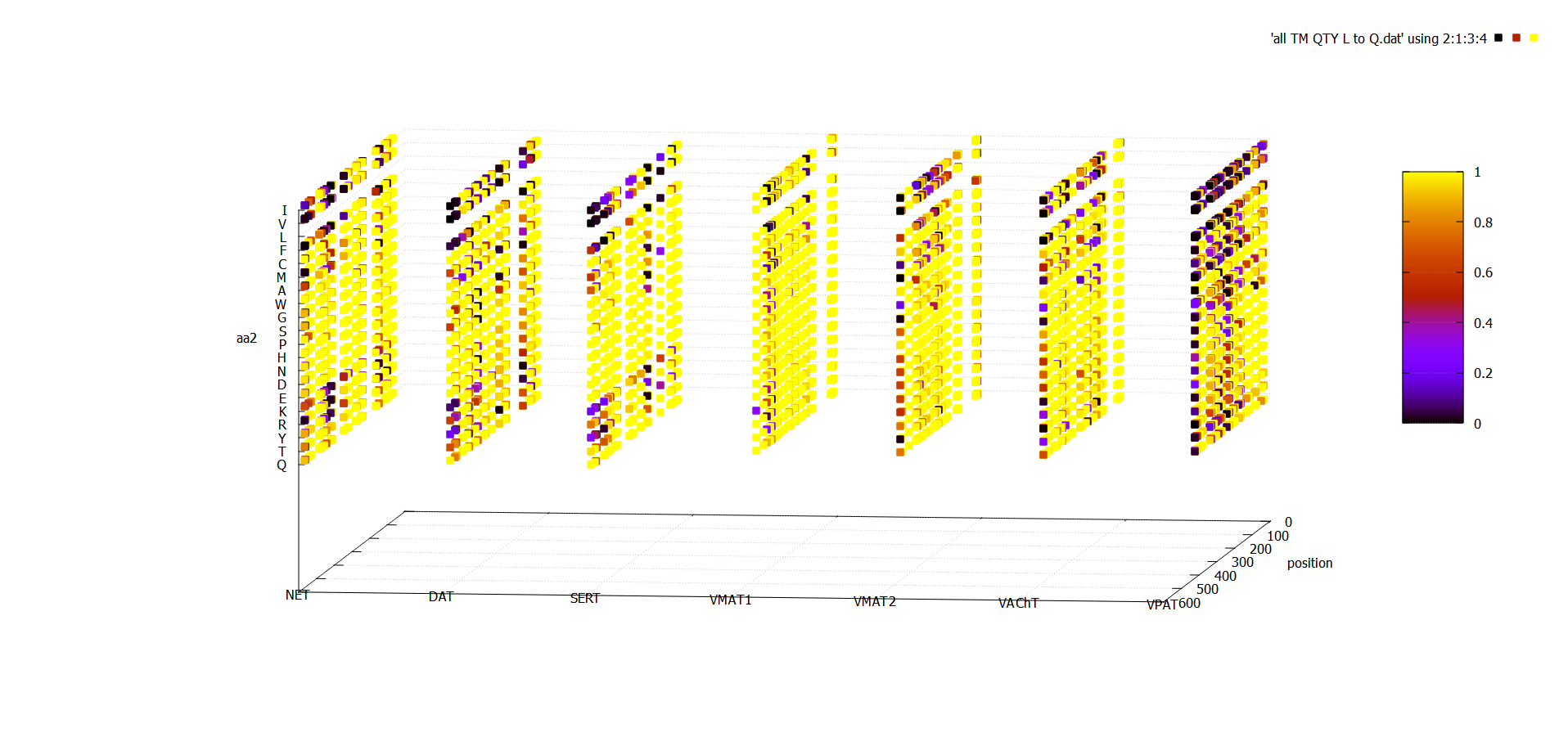


**Figure S7. Predicted effects of variations at the L amino acid residue located in the TM segments of seven monoamine transporters.** The names of the transporters are listed on the x-axis. The z-axis indicates the position of the substitution within the native protein sequence, while the color scale represents the PolyPhen-2 predicted effect of the substitution, ranging from benign (0.0) to damaging (1.0). The y-axis indicates the second amino acid that replaced the wild-type (I) amino acid residue.


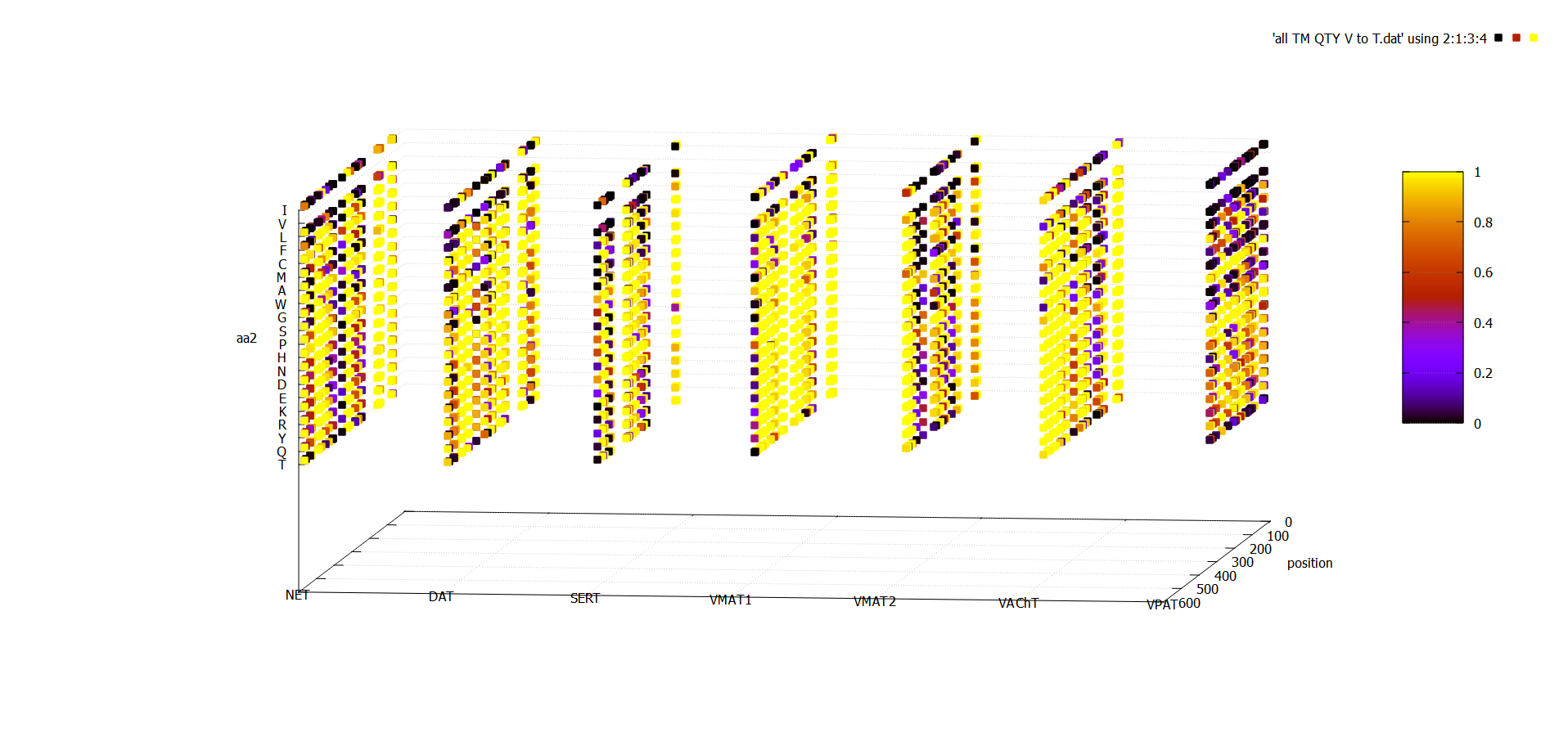


**Figure S8. Predicted effects of variations at the V amino acid residue located in the TM segments of seven monoamine transporters.** The names of the transporters are listed on the x-axis. The z-axis indicates the position of the substitution within the native protein sequence, while the color scale represents the PolyPhen-2 predicted effect of the substitution, ranging from benign (0.0) to damaging (1.0). The y-axis indicates the second amino acid that replaced the wild-type (I) amino acid residue.


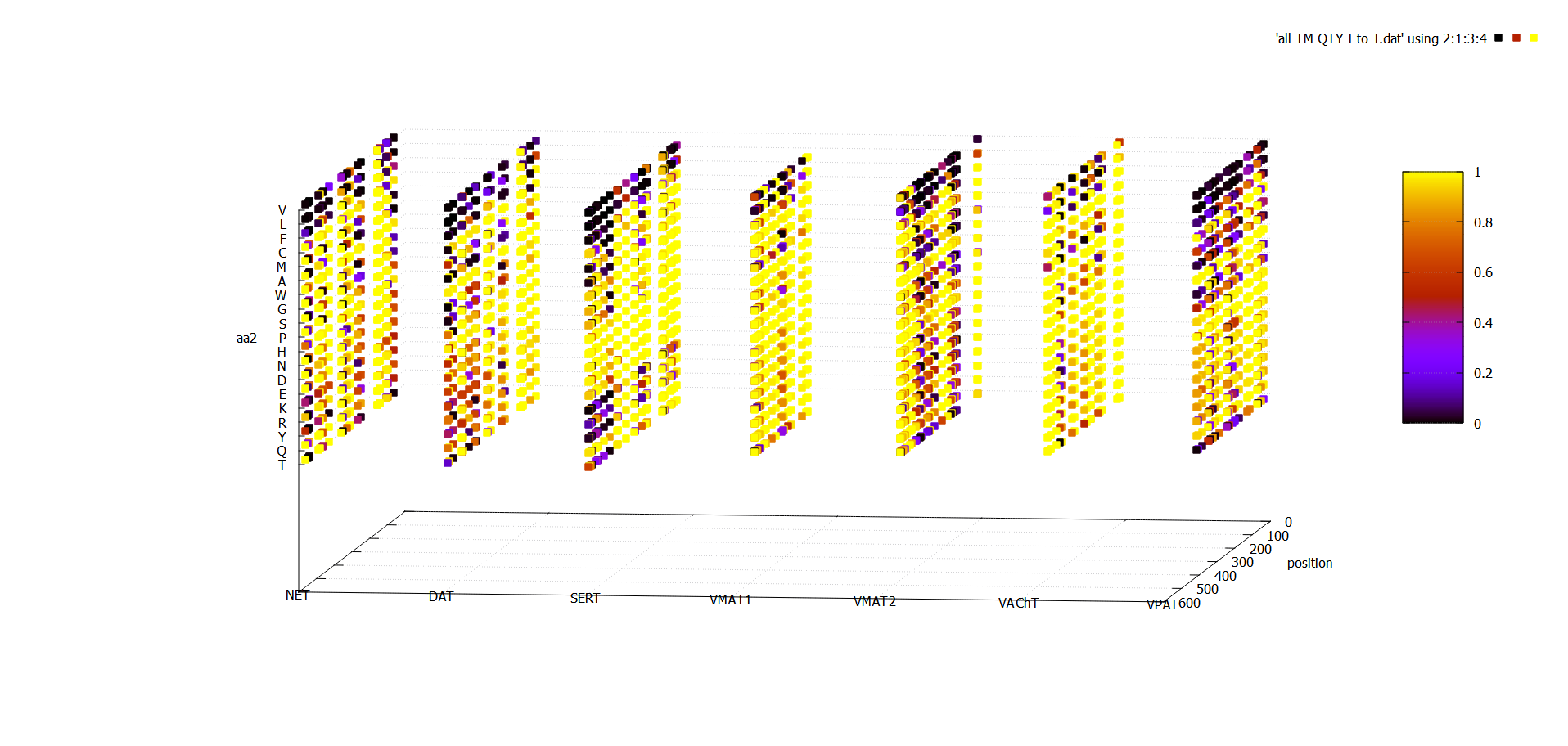


**Figure S9. Predicted effects of variations at the I amino acid residue located in the TM segments of seven monoamine transporters.** The names of the transporters are listed on the x-axis. The z-axis indicates the position of the substitution within the native protein sequence, while the color scale represents the PolyPhen-2 predicted effect of the substitution, ranging from benign (0.0) to damaging (1.0). The y-axis indicates the second amino acid that replaced the wild-type (I) amino acid residue.


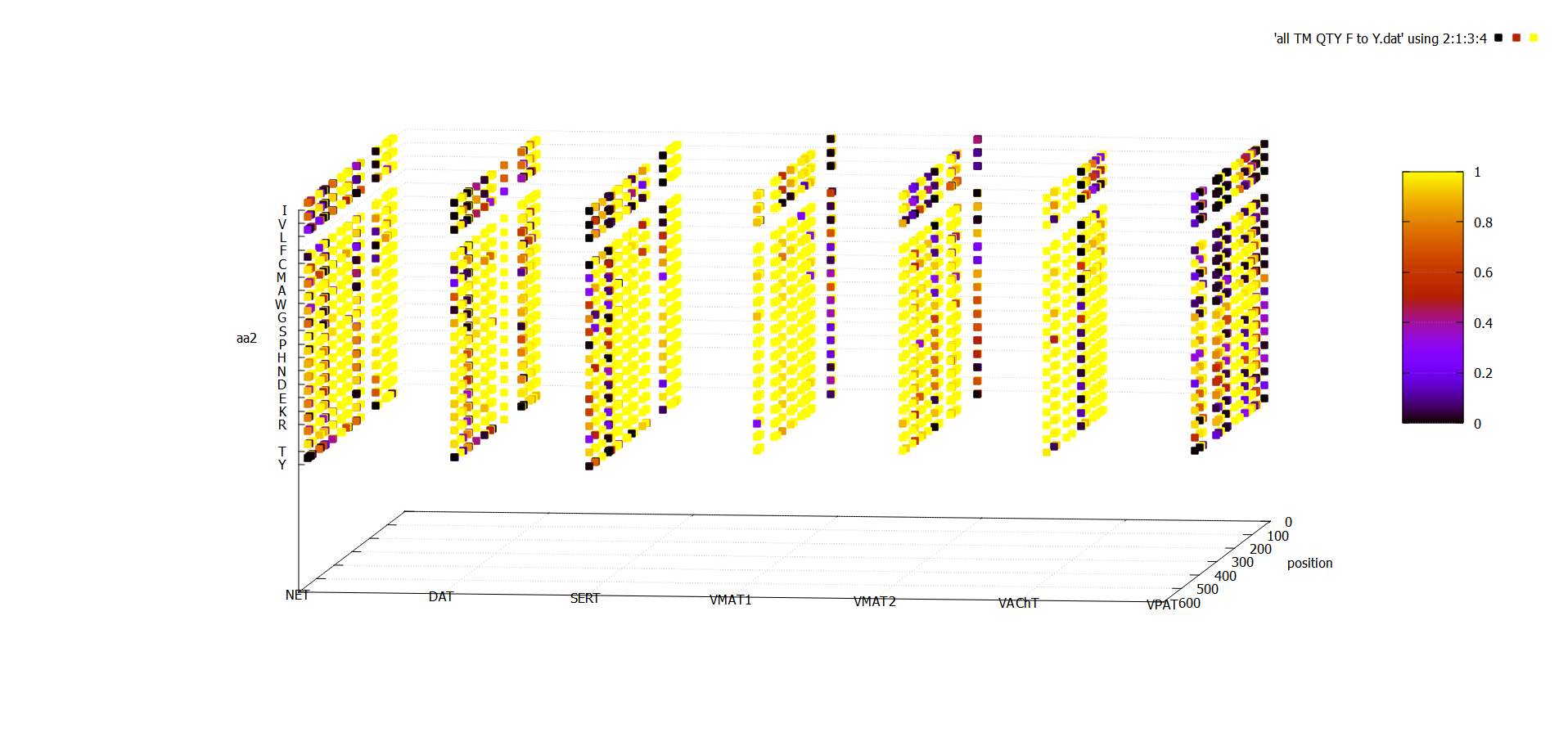


**Figure S10. Predicted effects of variations at the F amino acid residue located in the TM segments of seven monoamine transporters.** The names of the transporters are listed on the x-axis. The z-axis indicates the position of the substitution within the native protein sequence, while the color scale represents the PolyPhen-2 predicted effect of the substitution, ranging from benign (0.0) to damaging (1.0). The y-axis indicates the second amino acid that replaced the wild-type (I) amino acid residue.


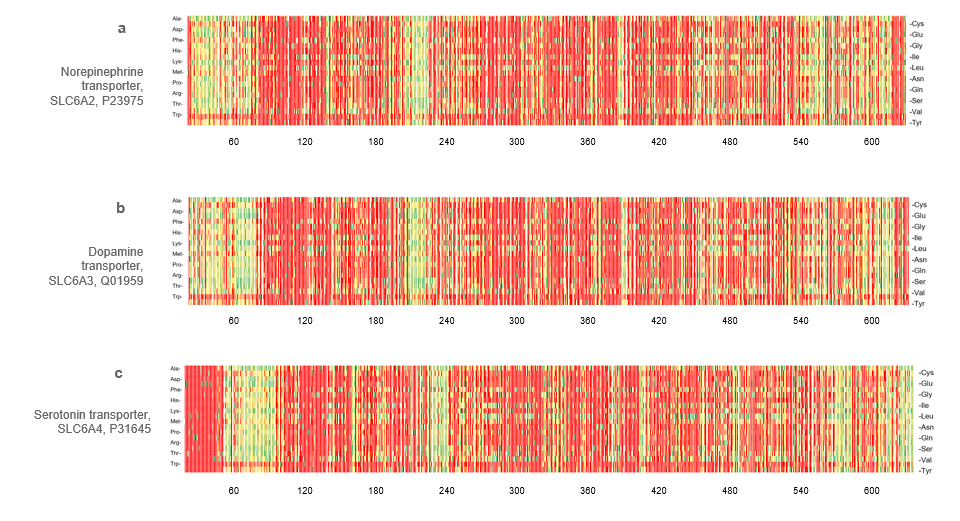


**Figure S11. Mutation visualizations of NET, DAT, and SERT.** Predicted probabilities of all possible mutations at each amino acid residue of 3 monoamine transporters: NET **(a)**, DAT **(b)**, SERT **(c)**. The mutations are color-coded as green for neutral or red for pathological. The SIFT predictions for entire proteins are color coded from red (deleterious) to green (neutral). Amino acids substitutions with probabilities < .05 are predicted to be deleterious (red).


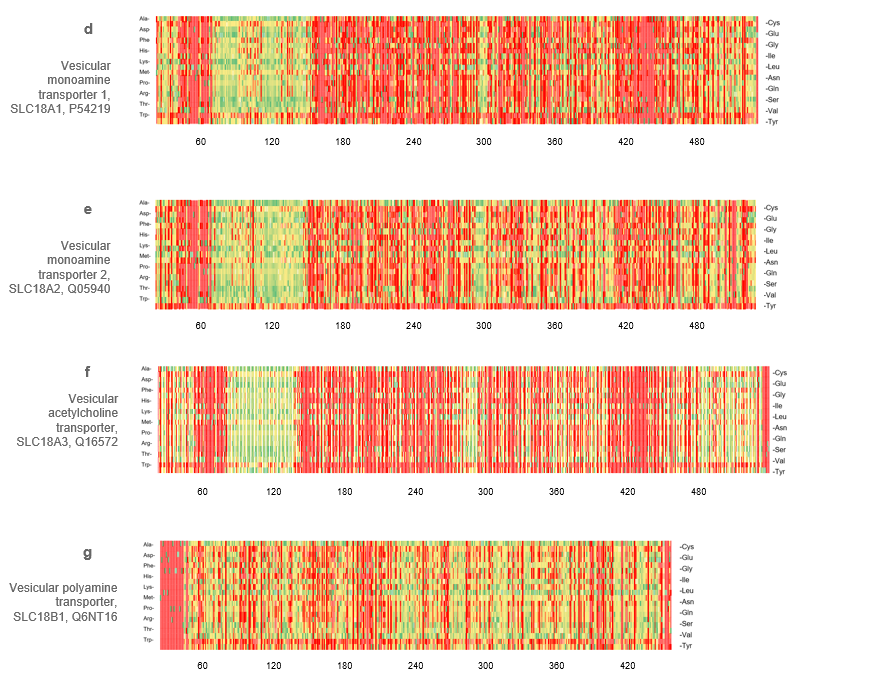


**Figure S12**. **Mutation visualizations of VMATs, VAChT, and VPAT**. Predicted probabilities of all possible mutations at each amino acid residue of 4 transporters: VMAT1 **(a)**, VMAT2 **(b)**, VAChT **(c)**, VPAT **(d)**. The mutations are color-coded as green for neutral or red for pathological. The SIFT predictions for entire proteins are color coded from red (deleterious) to green (neutral). Amino acids substitutions with probabilities < .05 are predicted to be deleterious (red).


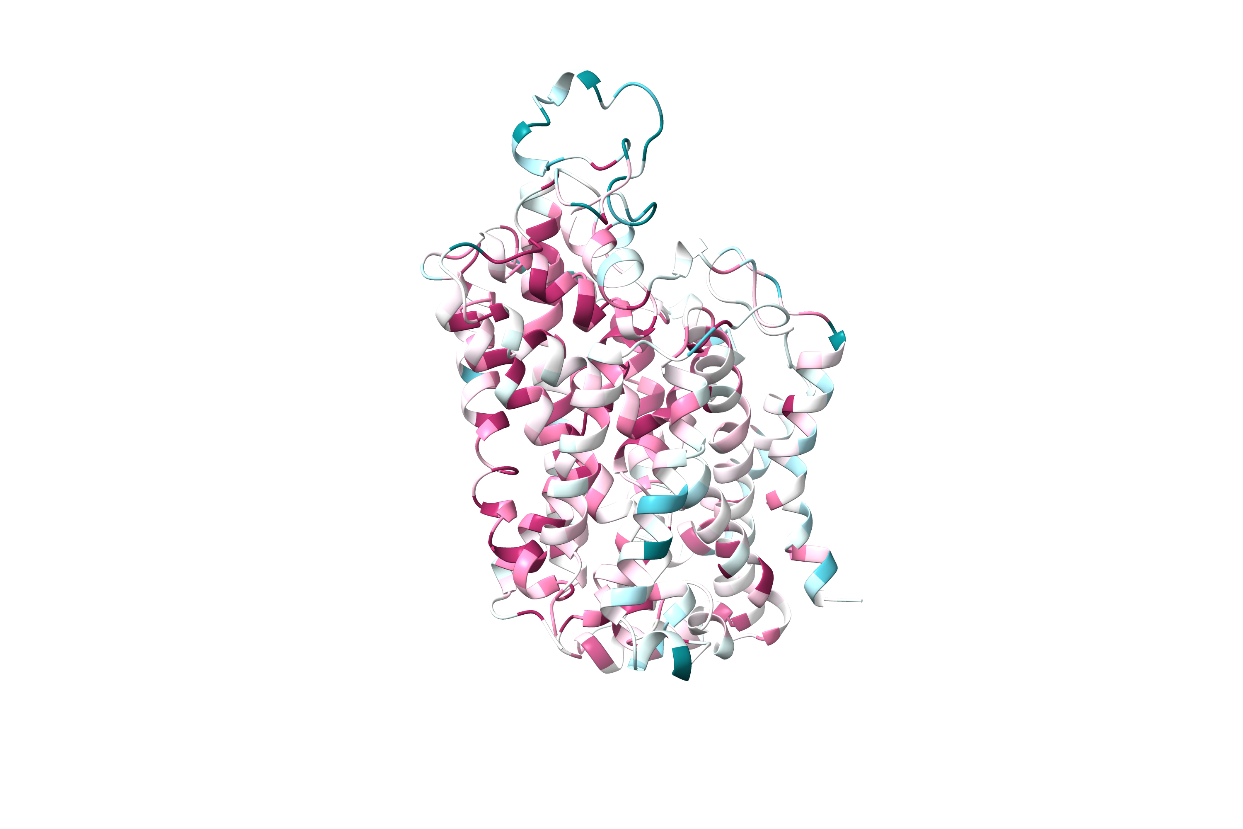

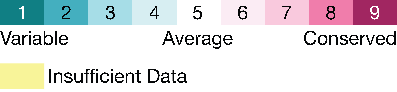

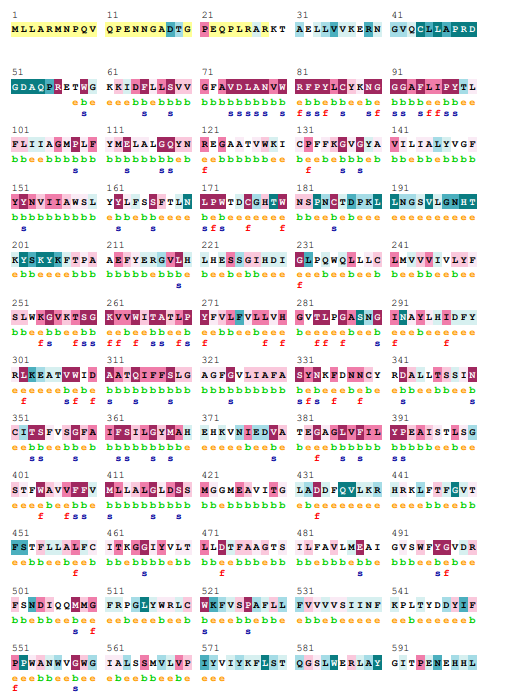

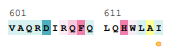


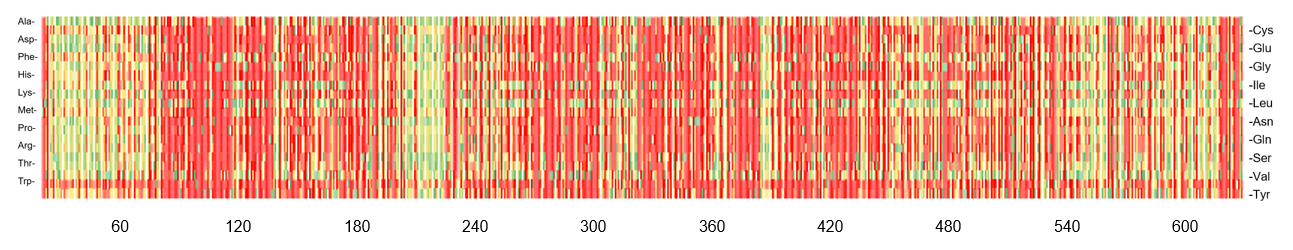


**Figure S13. Norepinephrine transporter (NET) evolutionary conservation profiles and mutation visualizations.** Evolutionary conservation grades of each amino acid residue predicted by ConSurf server; visualized by the color-coding scheme of nine colors, ranging from turquoise (variable) through white (average) through burgundy (conserved) represents conservation grades 1 to 9, in order of increasing conservation (1= Variable, 5= Average, 9= Conserved). Conservation grades were calculated for the source amino acid sequence and the corresponding Alphafold2 predicted native structure. For clarity, the N- and C-termini and large loops, which are often not resolved in experimental structures, were deleted. At the bottom of the figure, the predicted pathologies of all possible NET mutations are displayed. The mutations are color-coded as green for neutral or red for pathological.


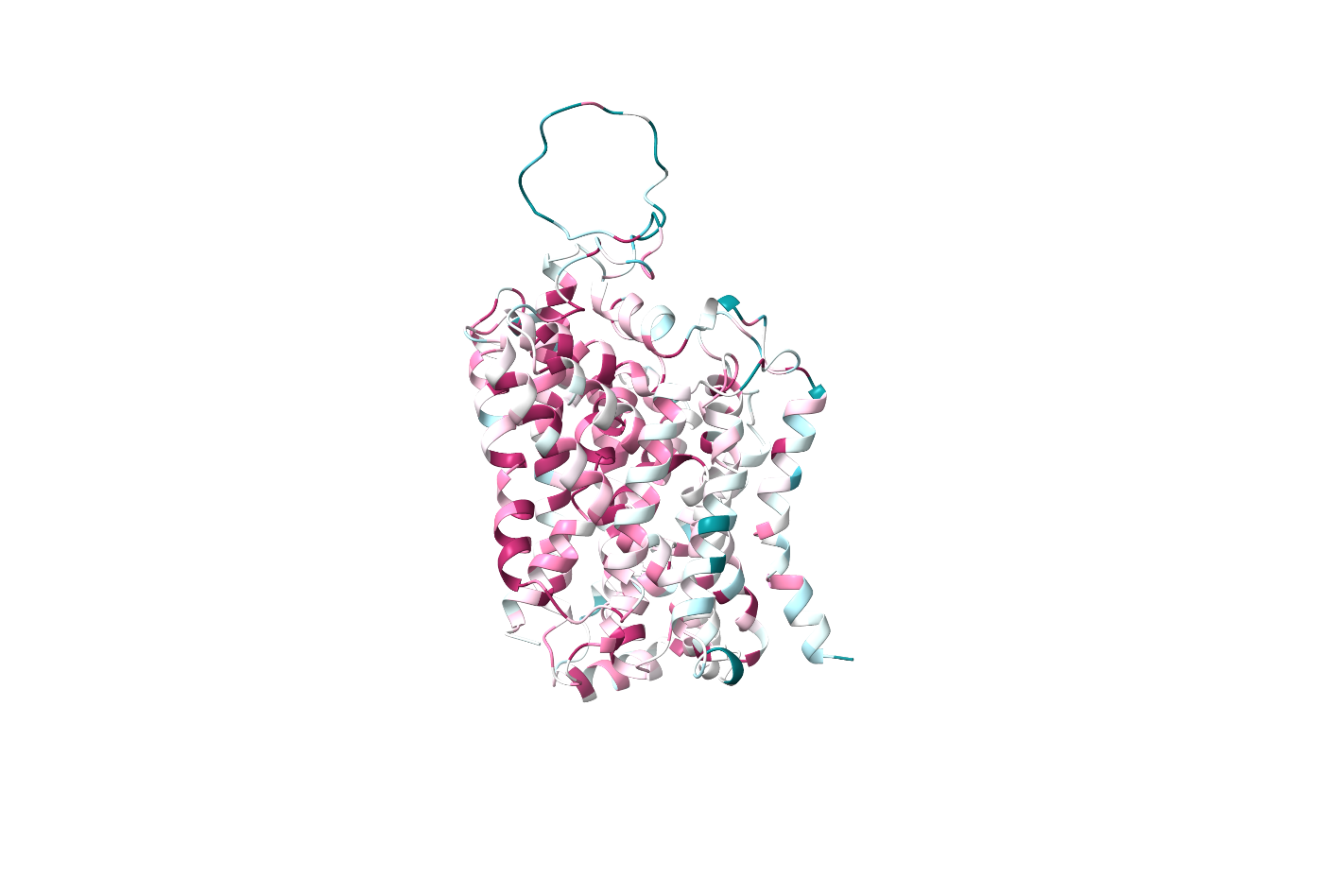

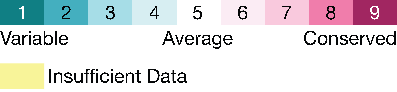

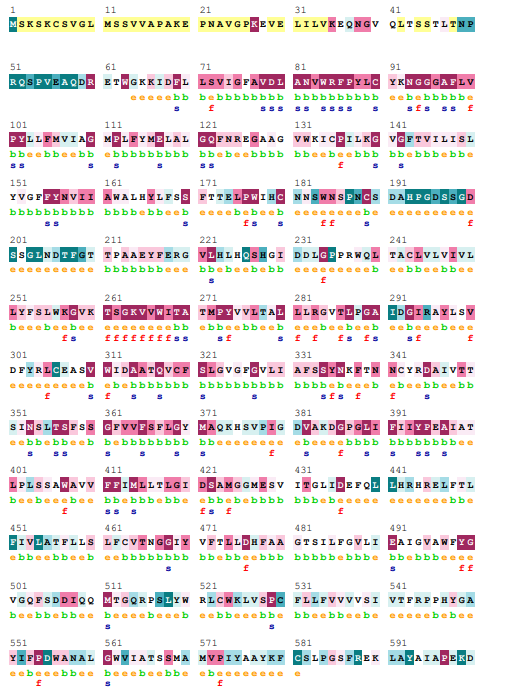

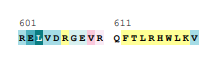


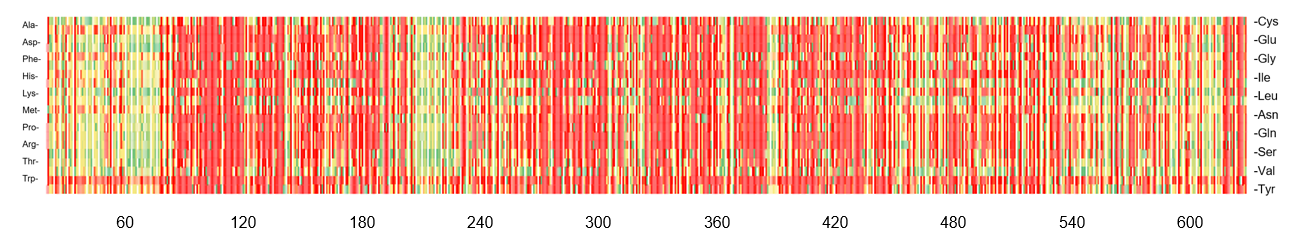


**Figure S14. Dopamine transporter (DAT) evolutionary conservation profiles and mutation visualizations.** Evolutionary conservation grades of each amino acid residue predicted by ConSurf server; visualized by the color-coding scheme of nine colors, ranging from turquoise (variable) through white (average) through burgundy (conserved) represents conservation grades 1 to 9, in order of increasing conservation (1= Variable, 5= Average, 9= Conserved). Conservation grades were calculated for the source amino acid sequence and the corresponding Alphafold2 predicted native structure. For clarity, the N- and C-termini and large loops, which are often not resolved in experimental structures, were deleted. At the bottom of the figure, the predicted pathologies of all possible DAT mutations are displayed. The mutations are color-coded as green for neutral or red for pathological.


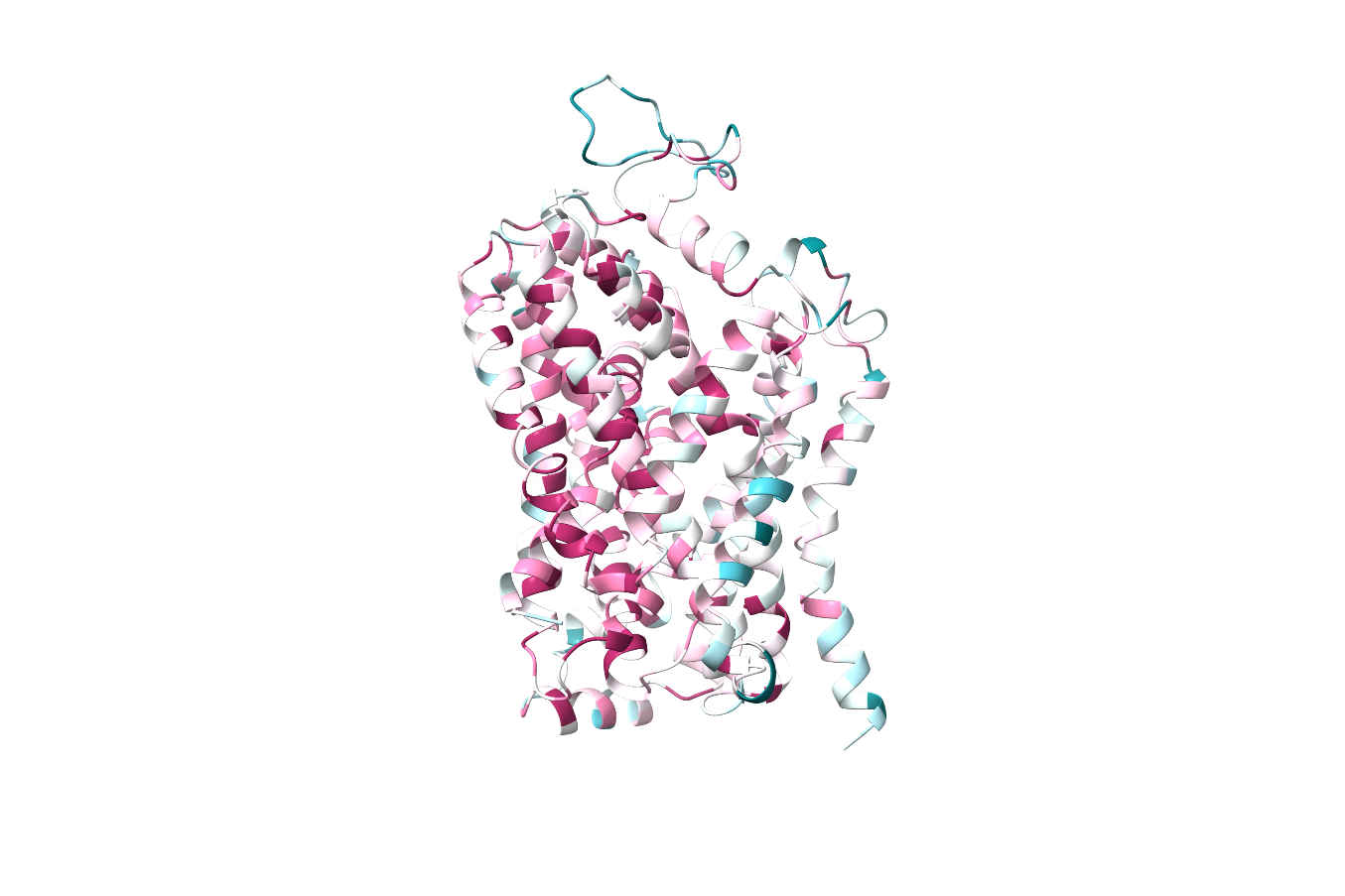

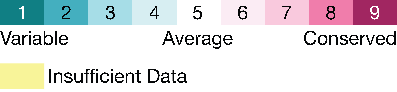

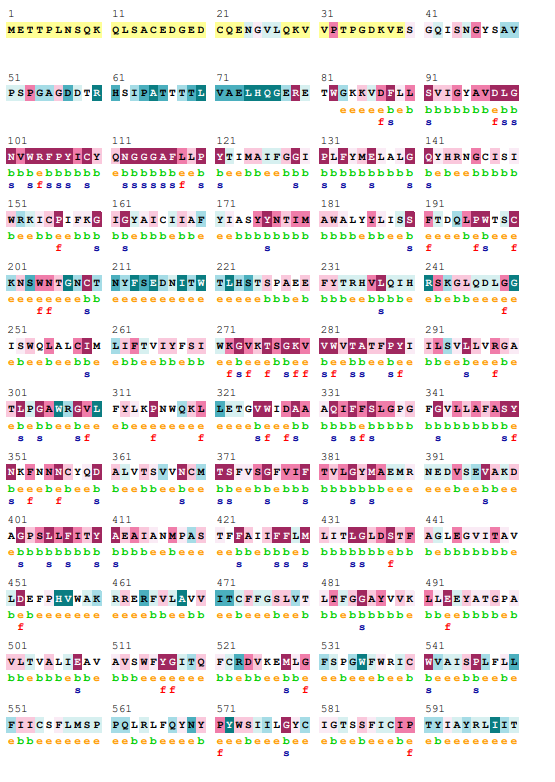

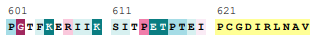


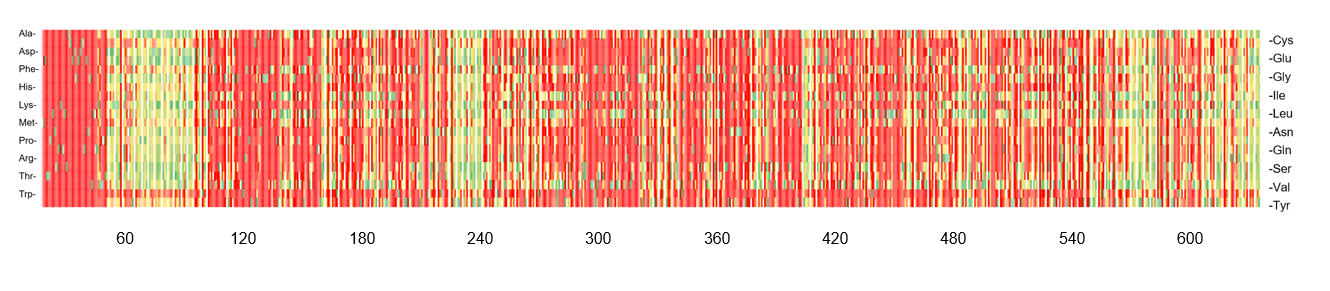


**Figure S15. Serotonin transporter (SERT) evolutionary conservation profiles and mutation visualizations.** Evolutionary conservation grades of each amino acid residue predicted by ConSurf server; visualized by the color-coding scheme of nine colors, ranging from turquoise (variable) through white (average) through burgundy (conserved) represents conservation grades 1 to 9, in order of increasing conservation (1= Variable, 5= Average, 9= Conserved). Conservation grades were calculated for the source amino acid sequence and the corresponding Alphafold2 predicted native structure. For clarity, the N- and C-termini and large loops, which are often not resolved in experimental structures, were deleted. At the bottom of the figure, the predicted pathologies of all possible SERT mutations are displayed. The mutations are color-coded as green for neutral or red for pathological.


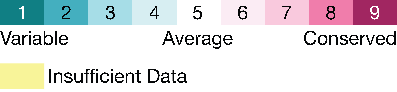

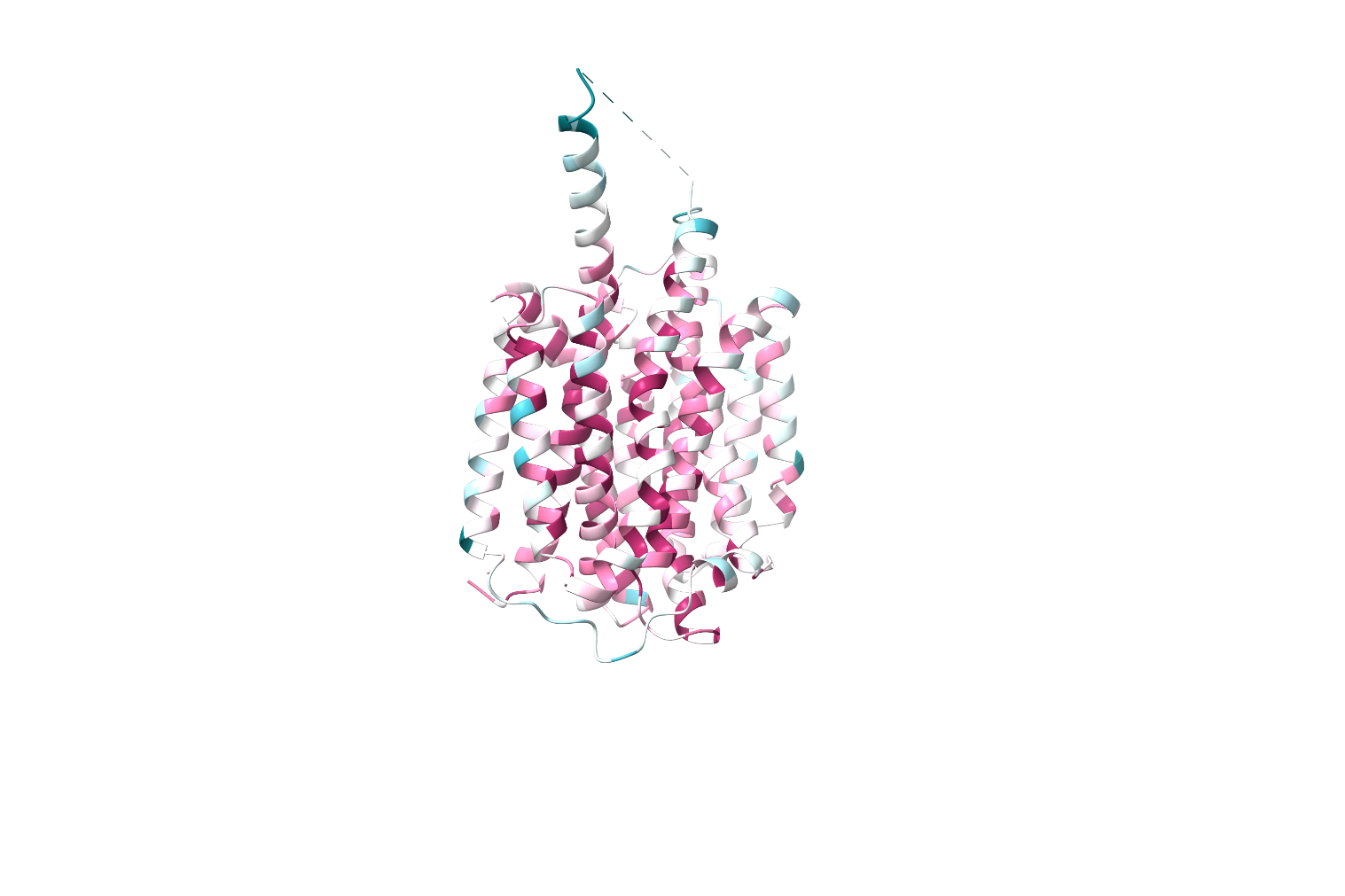

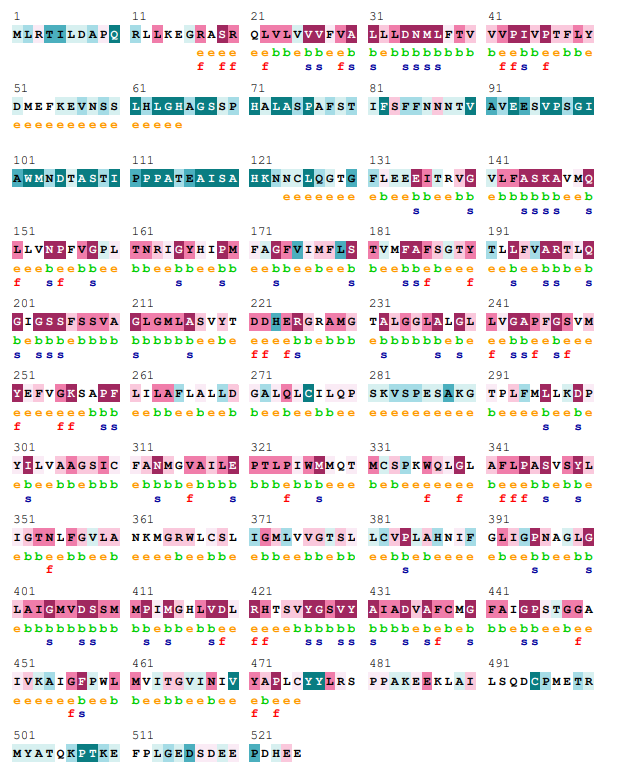


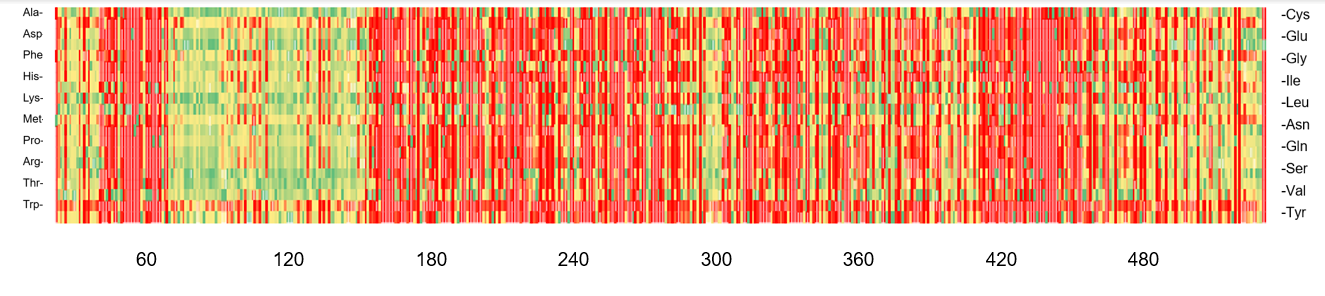


**Figure S16. Vesicular monoamine transporter 1 (VMAT1) evolutionary conservation profiles and mutation visualizations.** Evolutionary conservation grades of each amino acid residue predicted by ConSurf server; visualized by the color-coding scheme of nine colors, ranging from turquoise (variable) through white (average) through burgundy (conserved) represents conservation grades 1 to 9, in order of increasing conservation (1= Variable, 5= Average, 9= Conserved). Conservation grades were calculated for the source amino acid sequence and the corresponding Alphafold2 predicted native structure. For clarity, the N- and C-termini and large loops, which are often not resolved in experimental structures, were deleted. At the bottom of the figure, the predicted pathologies of all possible VMAT1 mutations are displayed. The mutations are color-coded as green for neutral or red for pathological.


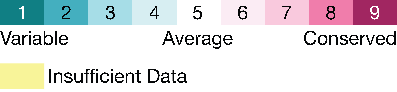

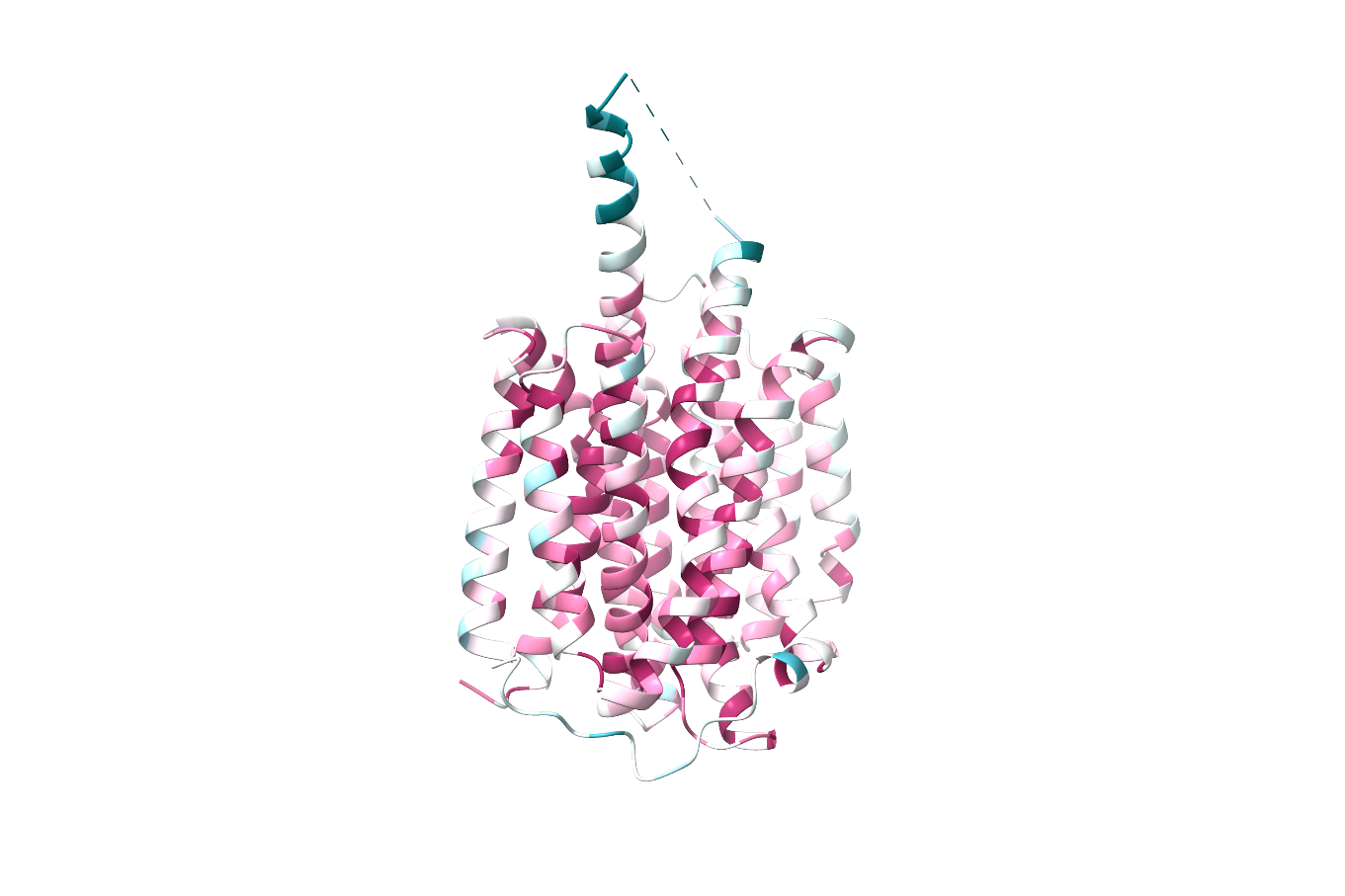

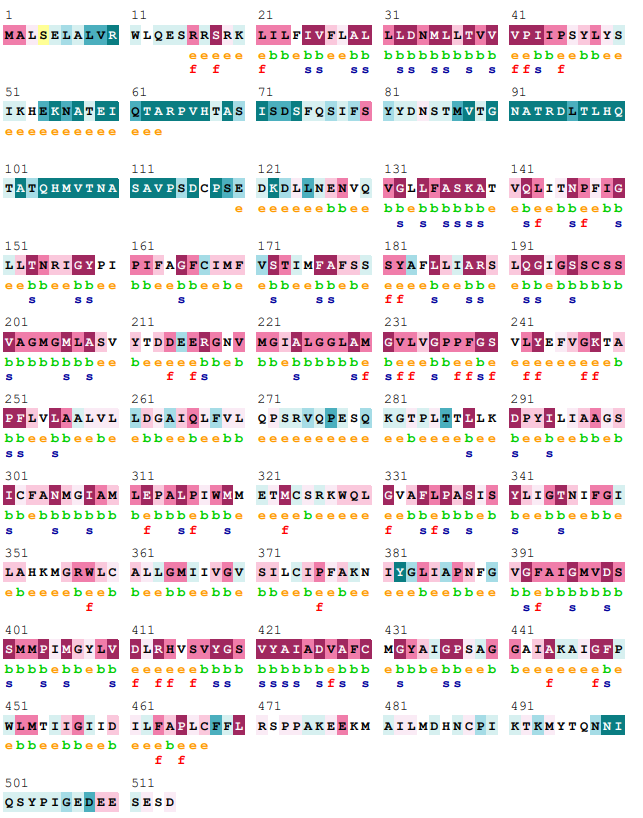


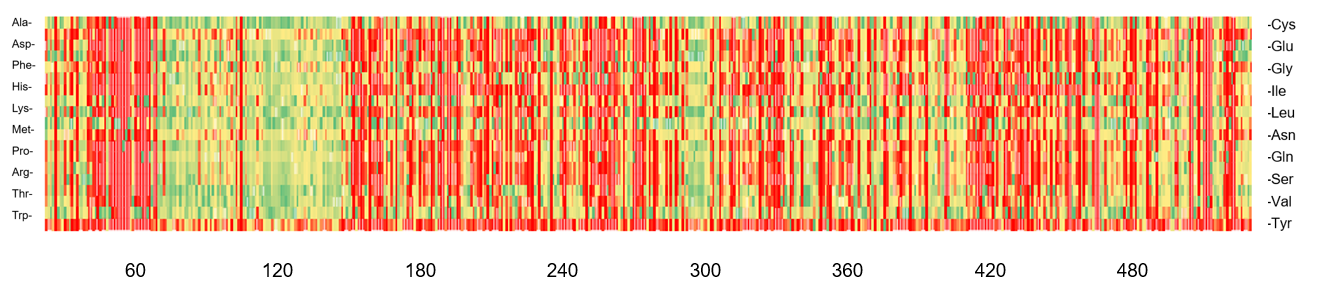


**Figure S17. Vesicular monoamine transporter 2 (VMAT2) evolutionary conservation profiles and mutation visualizations.** Evolutionary conservation grades of each amino acid residue predicted by ConSurf server; visualized by the color-coding scheme of nine colors, ranging from turquoise (variable) through white (average) through burgundy (conserved) represents conservation grades 1 to 9, in order of increasing conservation (1= Variable, 5= Average, 9= Conserved). Conservation grades were calculated for the source amino acid sequence and the corresponding Alphafold2 predicted native structure. For clarity, the N- and C-termini and large loops, which are often not resolved in experimental structures, were deleted. At the bottom of the figure, the predicted pathologies of all possible VMAT2 mutations are displayed. The mutations are color-coded as green for neutral or red for pathological.


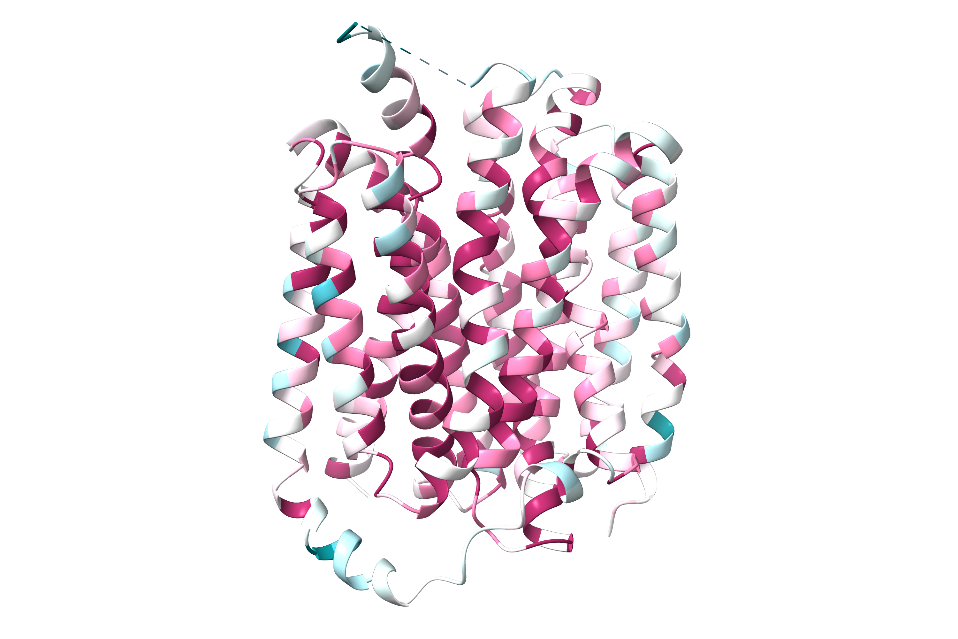

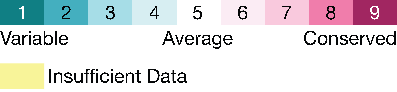

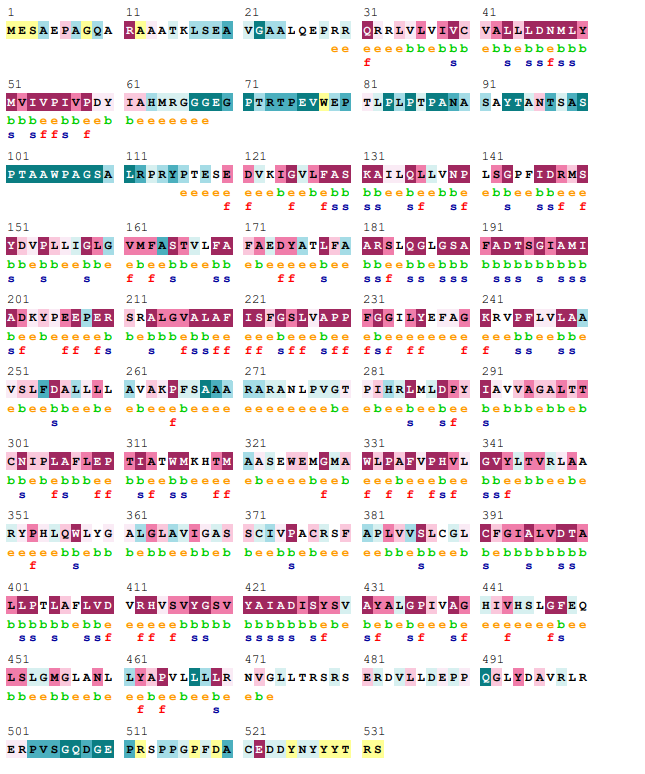


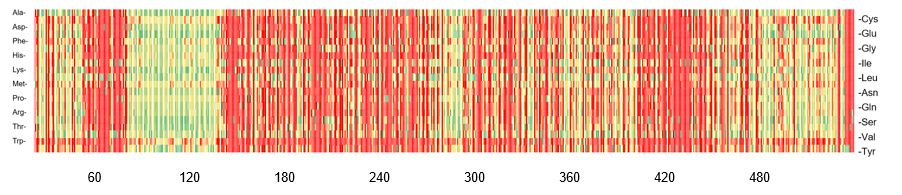


**Figure S18. Vesicular acetylcholine transporter (VAChT) evolutionary conservation profiles and mutation visualizations.** Evolutionary conservation grades of each amino acid residue predicted by ConSurf server; visualized by the color-coding scheme of nine colors, ranging from turquoise (variable) through white (average) through burgundy (conserved) represents conservation grades 1 to 9, in order of increasing conservation (1= Variable, 5= Average, 9= Conserved). Conservation grades were calculated for the source amino acid sequence and the corresponding Alphafold2 predicted native structure. For clarity, the N- and C-termini and large loops, which are often not resolved in experimental structures, were deleted. At the bottom of the figure, the predicted pathologies of all possible VAChT mutations are displayed. The mutations are color-coded as green for neutral or red for pathological.


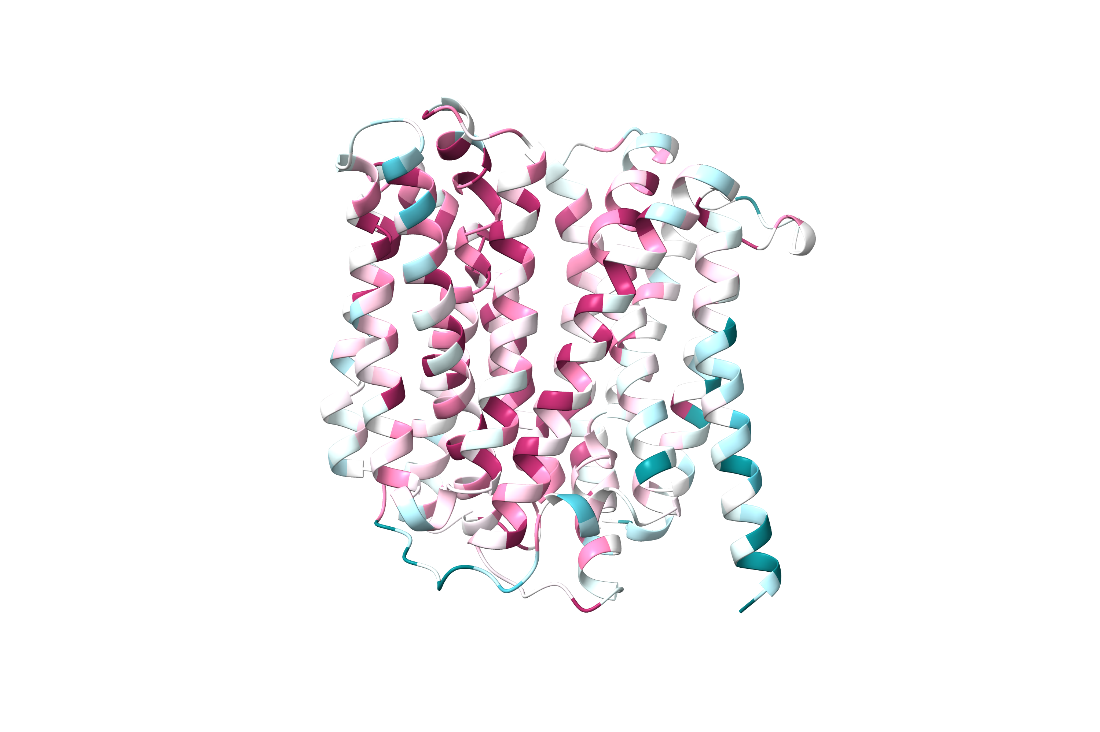

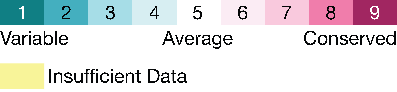

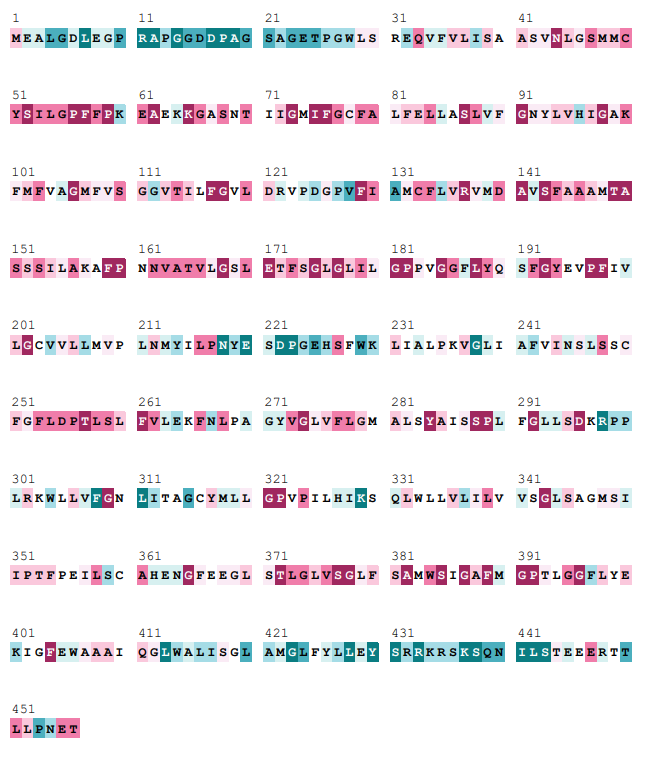

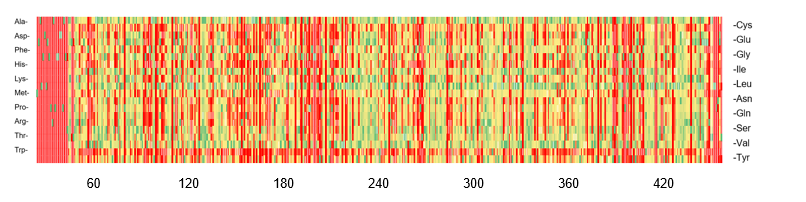


**Figure S19. Vesicular polyamine transporter (VPAT) evolutionary conservation profiles and mutation visualizations**. Evolutionary conservation grades of each amino acid residue predicted by ConSurf server; visualized by the color-coding scheme of nine colors, ranging from turquoise (variable) through white (average) through burgundy (conserved) represents conservation grades 1 to 9, in order of increasing conservation (1= Variable, 5= Average, 9= Conserved). Conservation grades were calculated for the source amino acid sequence and the corresponding Alphafold2 predicted native structure. For clarity, the N- and C-termini and large loops, which are often not resolved in experimental structures, were deleted. At the bottom of the figure, the predicted pathologies of all possible VPAT mutations are displayed. The mutations are color-coded as green for neutral or red for pathological.


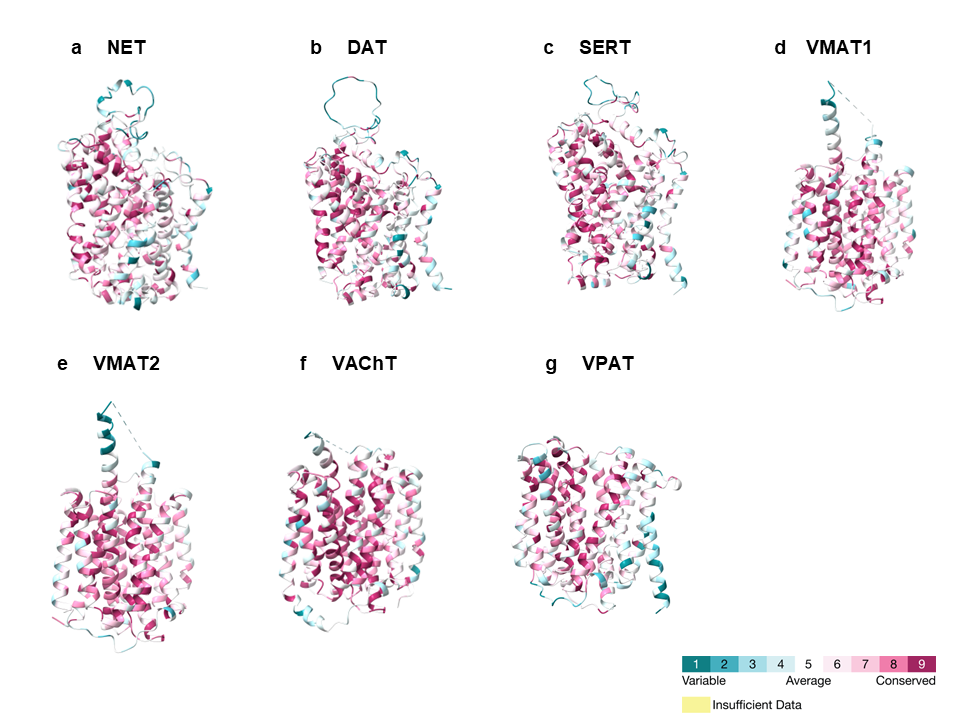


**Figure S20**. **Evolutionary conservation profiles of the 7 native monoamine transporters** **that were predicted by AlphaFold2.** The predicted native structures and their residues colored by evolutionary conservation grades. The number of residues that have more than average conservation grade was calculated as follows: ~60.6% for NET (313/517), ~60.2% for DAT (312/518), ~61.0% for SERT (316/518), ~67.8% for VMAT1 (272/401), ~73.2% for VMAT2 (290/396), ~70.9% for VAChT(292/398), ~58.8% for VPAT (238/405). Please note that he higher conservations of VMATs and VAChT are correlated with their higher ratio of transmembrane residues (Figure S2). VPAT is involved in the transport of polyamines, which are not neurotransmitters, so the selective pressure to conserve VPAT may not be as high. VPAT was also found to be less sensitive to mutations than the other six transporters (Please see Figure S10 and Figure S11).
