# Supplementary material for: Structural bioinformatics studies of serotonin, dopamine and norepinephrine transporters and their AlphaFold2 predicted water-soluble QTY variants and uncovering the natural mutations of L->Q, I->T, F->Y and Q->L, T->I and Y->F
Source: PLoS One. 2024 Mar 22;19(3):e0300340. doi: 10.1371/journal.pone.0300340 (PMC10959339; doi:10.1371/journal.pone.0300340)
Supplement: Attachment — Submitted filename: PONE-D-23-35512.docx [file pone.0300340.s002.docx]

**Comments: Minor Revision**

This study applies a water solubilization design method called QTY code to monoamine transporters, computationally characterizing some properties of the designed proteins and investigating the relationship between natural mutations in these monoamine transporters and the QTY code. These findings are intriguing and hold some significance for the study on monoamine transporters and the QTY code; however, there are some issues in the manuscript should be addressed by the authors.

**Specific comments:**

1. Abstract：*While their hydrophobic surfaces significantly reduced, this change did not cost a structural alteration.*

This statement tends to be too assertive. However, after numerous sequence variations, there must be structural changes, to varying degrees.

2. Abstract：*Accompanied by similarities of QTY-code pairs, our study unrevealed multiple QTY and reverse QTY variations in genomic databases.*

Please provide a more explicit explanation regarding the specific aspects of similarity. Additionally, the use of the term 'QTY-code pair' in the abstract is also confusing.

3. The structure of Introduction section needs improvement. The current manuscript presents introductions to various aspects (transporter, QTY code, transporter mutation) in a somewhat confusing manner, with much interweaving. This may make it challenging for readers to quickly and clearly grasp the background knowledge on these topics.
4. The order of figures needs to be revised, as Figure S19 appears earliest and should not be the last supplementary figure.
5. Page 4: *The QTY code substitutes four hydrophobic amino acids (Leucine, Isoleucine, Valine, and Phenylalanine) with hydrophilic amino acids (Glutamine = Q, Threonine = T, and Tyrosine = Y)*

The content related to the QTY code has already been introduced earlier; no need to repeat. The meanings of single letters also do not need to be redundantly explained.

6. Page 4: *The highly predicted Local Distance Difference Test (pLDDT) scores of the predictions are reassuring. Indicating greater confidence in predicted protein structures [33].*

This sentence needs to be rewritten.

7. In Page 4, it is mentioned that the pLDDT score is used to assess the quality of AlphaFold2 predictions. It is quite perplexing that the authors have not provided the pLDDT scores for the QTY variants and their distribution in the structures.
8. In Page 4, a significant portion is dedicated to introduce hydrophilic and hydrophobic alpha-helices. It is recommended to place this information after the results and reduce its length.
9. Table 2 consists of only one row and does not need to be presented as a separate table.
